# Supplementary material for: Effect of balanced energy-protein supplementation during pregnancy and lactation on birth outcomes and infant growth in rural Burkina Faso: study protocol for a randomised controlled trial
Source: BMJ Open. 2021 Mar 24;11(3):e038393. doi: 10.1136/bmjopen-2020-038393 (PMC7993280; doi:10.1136/bmjopen-2020-038393)
Supplement: Supplementary data [file bmjopen-2020-038393supp002.pdf]

1. PARTICIPANT INFORMATION SHEET

THE EFFECT OF BALANCED ENERGY-PROTEIN SUPPLEMENTATION DURING PREGNANCY AND LACTATION ON BIRTH OUTCOMES AND INFANT GROWTH IN RURAL BURKINA FASO

Coordinating Investigator :	Prof. Dr. Patrick Kolsteren
Principal Investigator:	Dr. Laeticia Celine TOE
Sponsor of the study:	Ghent University
Participant Number:	|__||__||__||__||__| _ |__||__|

Dear madam,

You are invited to participate in a study that wants to study the effect of providing a food supplement during pregnancy and lactation on the growth of your baby. The study is a collaboration between the Institut de Recherche en Sciences de la Santé (IRSS) and AfricSanté in Burkina Faso, Ghent University of Belgium and the International Food Policy Research Institute (IFPRI) in the USA. Before you decide to participate in this study, it is important that you read/ are read and explained to this form, because it explains your rights and our responsibilities to you. In this information and consent form, the purpose, examinations, advantages, risks and inconveniences related to this study are explained. The available alternatives and the right to withdraw your consent to participate at any time are also described below. No promises or guarantees can be made about the results of the study. You have the right to ask questions at any time, for example about the possible and/or known risks related to this study.

PURPOSE AND DESCRIPTION OF THE STUDY

This research study will provide more evidence and insight into how we can improve pregnancy outcomes , birthweight of babies and the growth of newborns. With this study, we aim to assess whether the provision of the food supplement under investigation can improve the growth of fetuses during pregnancy and that of infants during their first 6 months of life. In addition, we will evaluate how the food supplements that are offered to you improve the nutritional quality of the human milk produced for your child, as well as your and your child’s body composition.

We will thus compare the effect of 2 different approaches. One is to give you a tablet during your pregnancy and with the other approach participants will also receive a food supplement next to the tablet. The tablet is what you normally received for any pregnancy according to the guidelines of the ministry of health of Burkina Faso. We will provide the supplement for the entire duration of your pregnancy. After birth some mothers will continue to receive a food supplement for six months. Who receives what is entirely determined by chance. In total, 1776 pregnant women living in the catchment area of the health centers of de Boni, Dohoun, Dougmato II, Karaba, Kari and Koumbia will be recruited to participate in this study.

Vanslambrouck K, *et al. BMJ Open* 2021; 11:e038393. doi: 10.1136/bmjopen-2020-038393

How the study is done

If you are confirmed pregnant and accept to participate in this study, the midwife will do a full medical check-up of you and your baby according to medical standards. She will also inform you about a good dietary practices for you during your pregnancy.
After the check-up we will provide you either with the tablets, or the food supplements.

Thereafter, you will receive a daily visit from the community health worker who will ask you to take the tablet or eat the food supplement. A ultrasound examination will be planned for you within 2 weeks after your first consultation and will be performed by the project doctor. We will also arrange for you regular check-ups of your pregnancy by the midwife or the medical doctor.

Examinations in the context of the study

If you accept to participate in the study and if you and your child meet all the conditions for participating in the study, the following tests and examinations will be performed:

- We will take your weight t and height and ask you your age at inclusion.
- The midwife will invite you for antenatal check-ups according to the guidelines of the ministry of health and the medical doctor will perform an echography of your baby. This is not invasive and has no known risks. It is like taking a picture of your baby. As part of the routine check-up we will take fingerpick blood to test whether you are anemic and give you treatment when necessary.
- We will also ask you questions about your mental wellbeing, e.g. how you are living your pregnancy and if something bothers you in your everyday life.
- After delivery we will again take fingerpick blood to test whether you are anemic and give you treatment when necessary.
- When you deliver we will weigh your baby and measure his/her length and head and chest circumferences.
- After birth we will visit you or ask you to come to the health center every month to have the weight and the length of you baby taken. At that time we will also ask you question on illnesses your child might have had in the previous week and on what he/she has been eating.
- We will also perform a finger/heel prick on your baby to see if he/she is anemic and a treatment will be provided if necessary
- Halfway through pregnancy in some of the participating women, we will ask you questions about what and how much they have eaten the day before the interview. This should take about half an hour.

When the doctor identifies a medical problem he will see to it that you receive the appropriate information and necessary treatment, and will refer you when necessary.

If your child suffers from a disease or undernutrition, he/she will be treated in the best way possible. In such case, your child will be referred to the local health center or the district hospital, for further physical examination by a medical doctor.

Voluntary participation

You participate entirely voluntarily in this study. You have the right to refuse to participate in the study. Your decision to participate or not in this study, or to stop your participation in this study will have no influence whatsoever on present and future medical consultations and possible treatments. You also have the right yourself to stop your participation in the study at any time, even after you have signed

this informed consent form. The withdrawal of your consent will not cause any disadvantage or loss of advantages / privileges.

RISKS AND INCONVENIENCES

Finger-prick blood will be taken from you at the first ante-natal consultation. You will experience a prick in your finger, however the prick is not invasive. Antiseptic measures will be taken to prevent any inflammation/ infection of your finger.

The food supplement used in this study is safe for you and your baby. The food supplement contains milk, sugar, oil, peanut butter and a mix of vitamins. Previous studies in Houndé that provided similar food supplements during pregnancy have not documented any complications during pregnancy or delivery. However, the supplement contains milk, which can cause bloating, flatulence and other digestive discomfort in some people. We encourage you to notify us, should you experience these effects, so we can take measures to insure you the best comfort possible.

All other investigation are routinely done as part of the follow-up of pregnancy.

Some of you will be asked to donate a small amount of breast milk at two time points during the study (between 1-2 months and 3-4 months age of your child). This will not diminish the amount of milk for your child or influence lactating performance. We will ask your permission for this donation again when the time comes.

In a random sample of all participants, we will assess body composition after delivery in the mother and the infant, using a special water that has been proven safe for such use. If you are chosen for that examination, we will provide detailed explanations of the procedure to you and a specific consent will be ask before we perform the assessment. Your privacy will be respected at all times

BENEFITS

We expect to show that taking food supplements during pregnancy and lactation will help children grow better and improve the quality of breastmilk. If this would be the case we will have the possibility to change policy to provide supplements to pregnant and lactating women.

PROTECTION OF YOUR PRIVATE LIFE

Your identity and your participation in this study will be treated strictly confidential. The specific information we obtain from you will not be shared with anybody, except the study investigators and partner institutes. Your identity remains secret since your personal information will only be designated by a unique participant number. Your name will not appear in any reports or publication resulting from this study. After the study is completed, you may request information about the study results. As soon as possible (maximum 5 years) after the study is completed, all personal information from participant shall be deleted from all databases to ensure complete anonymity. Those anonymized databases shall be shared with other researchers to advance research on mother and child health. This shall be done in strict accordance with international laws and regulations about privacy.

ETHICS COMMITTEE

Before starting, this study has been reviewed and approved by an independent Ethics Committee in Belgium, namely the Ethics Committee of the University Hospital in Ghent and it has been reviewed and approved by the Ethics Committee Centre Muraz in Burkina Faso. These committees also perform

continuous reviews of the study during its progression to make sure the study is carried out in the safest possible way.

COMPENSATIONS AND INCIDENTS THAT MAY OCCUR IN THIS STUDY

All costs related to sickness occurring during the study will be reimbursed to you. Investigators shall seek to provide a compensation and/or the best possible treatment in the event of damages/ injuries that may occur as a result of your participation in this study. For any other damages thought to be related to your participation in this study, and occuring after the study has been completed, participant may file a complaint to the relevant jurisdiction, which will treated in accordance with applicable laws in Burkina Faso.

CONTACT PERSONS IN CASE YOU HAVE QUESTIONS ABOUT THIS STUDY

If you have any questions concerning your participation in this study, or think you have been injured as a result of the study or have a reaction to the study food, inform the village health worker who visits you. S/he will bring you immediately in contact with the project coordinator or the project doctor. All village health workers have cell phones to contact these persons directly. You may also contact, now, during or after the study:

The principle investigator in Burkina Faso: Dr. Laeticia Celine Toe, Tel : +226 71 00 72 72
The president of the ethic committee: Dr. Adama Dembélé, Tel: +226 20 97 01 02
Or The local project Coordinator : Mr. Moctar Ouédraogo, Tel: +226 70 23 81 98
Or project medical doctor: Compaoré S. Anderson Casimir Tel : +226 70 57 51 02

Informed consent

Before you agree to participate in the informal consensus activity, you need to be aware that:

- This study was presented and cleared by the Ethical Review Board of the Ghent University and the ethics committee of Centre Muraz, Burkina Faso.
- This clearance is not to be taken as an obligation to take part in this study.
- Your participation is only voluntary and will be confirmed by signing this form. If you wish, you can withdraw from this study at any point, even after signing this form; you can withdraw by contacting the researcher (below) through email or telephone. You do not have to motivate or explain the decision of withdrawal. In case of withdrawal, your data already collected will be used to assess study outcomes.
- You can revise your answers to the questions if you wish so.
- Your input will be stored anonymously; researchers not involved in the data collection will not have access to your personal data and name. Anonymized databases shall be shared with other researchers for to advance research on maternal and child health.
- You can contact the researcher or the coordinator of the project at any time if you wish to obtain more information regarding this study
- There are very limited risks related to your participation in this study. Those are mentioned under the section " risks and inconveniences" of the information sheet. However, in accordance with the Directive concerning experiments on humans (07/05/2004), a civil liability insurance has been foreseen in the event of any injury or damage occurring and deemed due to your participation in this study.

Part intended only for the participant

I, the undersigned, ____ (name and surname) confirm that I have been informed about the MISAME-III clinical trial and that I have received a copy of the Participant Information Sheet and a copy of the consent form. I confirm that I have read and / or understood the four pages of the information sheet for participants. The study responsible gave me enough information about the conditions and duration of the study, and the possible risks and disadvantages. In addition, I had enough time to review the information and to ask questions, and I received satisfactory answers.

- I understand that I can terminate my participation in this study at any time after notifying the study responsible and this decision will not cause any inconvenience to me or my child.
- I am aware of the purposes for which the data is collected, processed and used in this study.
- I am ready to give information about my medical history and that of my child, or about any medication taken or participation in another study.
- I voluntarily consent to participate in this study and to cooperate with the required examinations and tests.
- I consent to my data being shared anonymously for the benefit of research and maternal and child health.
- I understand that auditors, employee representatives, the Commission for Medical Ethics or competent authorities may inspect my data in order to verify the information collected. By signing this document, I give permission for this control. At all times my privacy will be respected.
- I understand that the biological samples collected from me and / or my child will be sent to Europe or the USA for analysis, and this in strict respect of my confidentiality.
- I am aware that this study has been approved by an independent commission for medical ethics linked to the University Hospital of Ghent, examined by the Ethics Committee of the Center Muraz in Burkina Faso and that this study will be carried out in accordance with the guidelines Good Clinical Practice Guidelines and the Declaration of Helsinki, established for the protection of people participating in clinical trials.

Signature (or fingerprint) of the participant

____

In case of minority

Informed consent of the legal representative

I, the undersigned, ____ (name and surname), legal representative of ____ (name and surname) confirm that I have been informed about the MISAME-III clinical trial and that I have received a copy information sheet for participants and a copy of the consent form. I confirm that I have read and / or understood the 6 pages of the information sheet for participants. The study responsible gave me enough information about the conditions and duration of the study, the procedures, the advantages and possible disadvantages. In addition, I had enough time to review the information and to ask questions, and received satisfactory answers.

- I understand that I can withdraw my consent at any time after having informed the study managers and this decision will not cause any inconvenience to my child / wife / ward or to myself.
- I am aware of the purposes for which the data is collected, processed and used in this study.
- I voluntarily consent to my child / wife / ward participating in this study.
- I understand that auditors, employee representatives, the Commission for Medical Ethics or competent authorities may inspect their data in order to verify the information collected. By signing this document, I give permission for this control. At all times his privacy will be respected.

Date: | ___ | ___ | / | ___ | ___ | / | ___ | ___ | ___ | ___ | (dd / mm / yyyy)

Signature (or fingerprint) of the participant's legal representative

____

Part intended for the investigator

I, the undersigned, ____ confirm that I have informed, ____

(full name of the participant) and that she has:

Consent to participate in the study ☐

Refused to participate in the study ☐

Reason for refusal (mark not filled in if no reason provided)

____

____

Date | ___ | ___ | / | ___ | ___ | / | ___ | ___ | ___ | ___ | (dd / mm / yyyy)

Signature: ____

If the participant is unable to read and / or write, an impartial witness should be present during the consent discussion. After having read and explained the information sheet and the informed consent form to the participant. After she has verbally consented to her participation in the study, and has affixed her fingerprint, the witness should complete the name of the participant, add the date, and personally sign and date the consent form. By signing the consent form, the witness certifies that the information in the information sheet and the consent form have been precisely explained and understood by the participant and that the participant has freely given her consent.

Name and first name (s) of witness:

____

Signature of witness :

____

Date: | ___ | ___ | / | ___ | ___ | / | ___ | ___ | ___ | ___ | (dd / mm / yyyy)

Vanslambrouck K, *et al. BMJ Open* 2021; 11:e038393. doi: 10.1136/bmjopen-2020-038393

Contact Principal investigator

TOE Laeticia Celine
IRSS/DRO site Ecole Jamot
01 BP 545 Bobo-Dioulasso
Tél: +226 71 00 72 72
Email: cellaety@yahoo.fr

Contact of project medical doctor
Compaoré S. Anderson Casimir
Tel : +226 70 57 51 02
Email : discomp4523@gmail.com
