# Supplementary material for: Effect of balanced energy-protein supplementation during pregnancy and lactation on birth outcomes and infant growth in rural Burkina Faso: study protocol for a randomised controlled trial
Source: BMJ Open. 2021 Mar 24;11(3):e038393. doi: 10.1136/bmjopen-2020-038393 (PMC7993280; doi:10.1136/bmjopen-2020-038393)
Supplement: Author's manuscript [file bmjopen-2020-038393.draft_revisions.pdf]

BMJ Open

BMJ Open is committed to open peer review. As part of this commitment we make the peer review history of every article we publish publicly available.

When an article is published we post the peer reviewers' comments and the authors' responses online. We also post the versions of the paper that were used during peer review. These are the versions that the peer review comments apply to.

The versions of the paper that follow are the versions that were submitted during the peer review process. They are not the versions of record or the final published versions. They should not be cited or distributed as the published version of this manuscript.

BMJ Open is an open access journal and the full, final, typeset and author-corrected version of record of the manuscript is available on our site with no access controls, subscription charges or pay-per-view fees (http://bmjopen.bmj.com).

If you have any questions on BMJ Open's open peer review process please email info.bmjopen@bmj.com

THE EFFECT OF BALANCED ENERGY-PROTEIN SUPPLEMENTATION DURING PREGNANCY AND LACTATION ON BIRTH OUTCOMES AND INFANT GROWTH IN RURAL BURKINA FASO : STUDY PROTOCOL FOR A RANDOMIZED CONTROLLED TRIAL

Journal:	*BMJ Open*
Manuscript ID	bmjopen-2020-038393
Article Type:	Protocol
Date Submitted by the Author:	11-Mar-2020

Complete List of Authors:	Vanslambrouck, Katrien; University of Ghent, Food Technology, Safety and Health, Faculty of Bioscience Engineering<br>de Kok, Brenda; Ghent University, Department of Food Technology, Safety and Health, Faculty of Bioscience Engineering<br>Toe, Laeticia Celine; Ghent University, Department of Food Technology, Safety and Health, Faculty of Bioscience Engineering; Institut de Recherche en Sciences de la Sante<br>De Cock, Nathalie; Ghent University, Department of Food Technology, Safety and Health, Faculty of Bioscience Engineering<br>Ouedraogo, Moctar; AfricSanté<br>Ganaba, Rasmané; AfricSanté<br>Lachat, Carl; Ghent University, Department of Food Technology, Safety and Health, Faculty of Bioscience Engineering<br>Huybregts, Lieven; IFPRI, PHN division<br>Kolsteren, Patrick; Ghent University, Department of Food Technology, Safety and Health, Faculty of Bioscience Engineering

Keywords:	Nutrition < TROPICAL MEDICINE, Community child health < PAEDIATRICS, Protocols & guidelines < HEALTH SERVICES ADMINISTRATION & MANAGEMENT

SCHOLARONE™ Manuscripts

I, the Submitting Author has the right to grant and does grant on behalf of all authors of the Work (as defined in the below author licence), an exclusive licence and/or a non-exclusive licence for contributions from authors who are: i) UK Crown employees; ii) where BMJ has agreed a CC-BY licence shall apply, and/or iii) in accordance with the terms applicable for US Federal Government officers or employees acting as part of their official duties; on a worldwide, perpetual, irrevocable, royalty-free basis to BMJ Publishing Group Ltd ("BMJ") its licensees and where the relevant Journal is co-owned by BMJ to the co-owners of the Journal, to publish the Work in this journal and any other BMJ products and to exploit all rights, as set out in our licence.

The Submitting Author accepts and understands that any supply made under these terms is made by BMJ to the Submitting Author unless you are acting as an employee on behalf of your employer or a postgraduate student of an affiliated institution which is paying any applicable article publishing charge ("APC") for Open Access articles. Where the Submitting Author wishes to make the Work available on an Open Access basis (and intends to pay the relevant APC), the terms of reuse of such Open Access shall be governed by a Creative Commons licence – details of these licences and which Creative Commons licence will apply to this Work are set out in our licence referred to above.

Other than as permitted in any relevant BMJ Author's Self Archiving Policies, I confirm this Work has not been accepted for publication elsewhere, is not being considered for publication elsewhere and does not duplicate material already published. I confirm all authors consent to publication of this Work and authorise the granting of this licence.

For peer review only

[Title Page File]

Title:

THE EFFECT OF BALANCED ENERGY-PROTEIN SUPPLEMENTATION DURING PREGNANCY AND LACTATION ON BIRTH OUTCOMES AND INFANT GROWTH IN RURAL BURKINA FASO : STUDY PROTOCOL FOR A RANDOMIZED CONTROLLED TRIAL

Authors:

Vanslambrouck Katrien [a], de Kok Brenda [a], Toe Laeticia Celine [a c], De Cock Nathalie [a], Ouedraogo Moctar [d] Ganaba Rasmané [d], Lachat Carl [a], Huybregts Lieven [b], Kolsteren Patrick [a]

Authors institutional affiliations:

[a] Department of Food Technology, Safety and Health, Faculty of Bioscience Engineering, Ghent University, Ghent, Belgium

[b] Poverty, Health and Nutrition Division, International Food Policy Research Institute (IFPRI), 1201 I Street NW, Washington, DC 20006, USA

[c] Institut de Recherche en Sciences de la Santé (IRSS), Bobo-Dioulasso, Burkina Faso

[d] Africsanté, Bobo-Dioulasso, Burkina Faso

Word count :

3.763 words

Correspondence :

Katrien.Vanslambrouck@ugent.be

Other email addresses :

Brenda.deKok@UGent.be - LaeticiaCeline.Toe@UGent.be - nadecock@gmail.com

obmoctar@gmail.com – rganaba@hotmail.com - Carl.Lachat@UGent.be – L.Huybregts@cgiar.org - Patrick.Kolsteren@UGent.be

For peer review only - http://bmjopen.bmj.com/site/about/guidelines.xhtml

[Main Text File]

Abstract

Introduction:

Adequate nutrition during pregnancy is crucial to both mother and child. Maternal malnutrition can be the cause of stillbirth or lead to poor birth outcomes such as preterm delivery and small-for-gestational-age new-borns. There is a possible positive effect of providing pregnant women a balanced energy and protein (BEP) food supplement but more evidence is needed. The MISAME III project is aiming to improve birth outcomes and infant growth by testing a BEP supplement during pregnancy and lactation in rural Burkina Faso. This paper presents the protocol.

Methods and analysis:

MISAME III is an individually randomized efficacy trial implemented in six rural health center catchments areas in the district of Houndé, Burkina Faso. Eligible pregnant women living in these areas and in the reproductive age range (between 15-40 years old) will be enrolled. Women will be randomly assigned to one of the four study groups: (i) prenatal intervention only, (ii) postnatal intervention only, (iii) prenatal and postnatal intervention or (iv) no prenatal or postnatal intervention. The intervention group will receive the BEP supplement and iron/folic acid (IFA) tablets while the control group will only receive the IFA tablets following the national health protocol. Consumption will be supervised by trained village women on a daily basis through home visits. The primary outcomes are small-for-gestational age at birth and length-for-age z-score at six months of age. Secondary outcomes will be measured at birth and during the first six months of the infants' life.

Ethics and dissemination:

MISAME III has been reviewed and approved by the University Hospital of Ghent University and the ethics committee of Centre Muraz, Burkina Faso. Informed consent will be obtained from participants. Results of the various studies will be published in relevant journals and shared with other researchers and public health institutions.

Trial registration number on Clinical Trial: NCT03533712.

Protocol v1

6/03/2020

For peer review only

Strengths and limitations of this study

- This trial will help to fill in the gap on how to improve birth outcomes in pregnant women in lower-and middle-income countries.
- Inclusion of formative research before the start of the efficacy trial will give us a better understanding on what product type to choose and the factors that determine compliance.
- Daily consumption of the BEP supplement and/or IFA tablets will be done under supervision during pregnancy and lactation.
- This study will be done in a rural district of Burkina Faso, generalizability of results might therefore be limited.
- Blinding the participants and all staff members will not be possible since the supplements are identifiable.

Keywords: Maternal nutrition, Dietary supplements, Pregnancy outcomes, Children's growth, Small for Gestational Age, Length for Age, Burkina Faso

For peer review only

Introduction

Pregnancy is a challenging period in the life of many women in lower- and middle-income countries (LMIC). Maternal mortality remains high and many neonates suffer from premature delivery and/or intra-uterine growth retardation both in length and in weight accumulation (1). An indicator to measure neonates growth is small-for-gestational age (SGA). SGA is defined as a birthweight below the 10th percentile of a standard optimal reference population for a given gestational age and sex (2). SGA is often caused by growth-restriction in the womb and has been associated with neonatal and post-neonatal mortality (2). It has also been linked to an increase in morbidity risks later in life, especially non-communicable diseases (3). SGA affected 23.3 million term children in LMIC in 2012 (4). Adequate nutrition during pregnancy is crucial for optimal maternal and newborn health (5, 6) and maternal malnutrition has been associated with fetal growth restriction (7). An adequate dietary balance is necessary to ensure sufficient energy intake for adequate growth of the fetus (8). Unfortunately, maternal undernutrition remains a big problem in regions across Sub-Saharan Africa and Asia (9, 10).

Several types of food supplements have been developed and evaluated over the past years. A positive effect of multiple-micronutrient supplements (MMS) during pregnancy on birth outcomes has been found in previous studies (11). Keats et al. concluded in their review that MMS during pregnancy gave a probable reduction in SGA and preterm births and can thus be used as a future guidance (11). According to a multi-country randomized controlled trial done in LMIC, a positive effect of lipid-based nutrient supplements on fetal growth-related birth outcomes can be seen starting before conception or starting during the first trimester (12). Moreover, the latest evidence indicates a possible positive effect of providing pregnant women a balanced energy and protein (BEP) food supplement (5, 13-15). In line with that evidence, the 2016 WHO antenatal care guidelines state that pregnant women in undernourished populations should receive - depending on the context - BEP supplements to reduce the risk of stillbirth and SGA (6). Researchers, however, still highlight the limited amount of evidence and a need to evaluate the effect of balanced supplements on birth outcomes, such as SGA (5, 13). Randomized trials of high quality and big sample sizes, especially in undernourished pregnant women are thus needed (13). Following this recommendation, a compositional guidance for a ready-to-use food supplement for pregnant women was developed by the Bill and Melinda Gates Foundation (BMGF) in 2016 (16).

For peer review only - http://bmjopen.bmj.com/site/about/guidelines.xhtml

The MISAME III (MIcronutriments pour la SAnté de la Mère et de l'Enfant) study hypothesizes that (i) providing women with a BEP supplement during pregnancy will decrease the incidence of SGA compared to the control group and that (ii) providing them with a BEP supplement during the postnatal period will increase growth in length by the age of six months compared to the control group.

Methods

MISAME III study design

The MISAME III project is an individually, randomized 2x2 factorial efficacy trial aiming to improve birth outcomes and infant growth in rural Burkina Faso by testing a BEP supplement during pregnancy and lactation **(figure 1**). At inclusion, pregnant women will individually and randomly be allocated to a pre-natal intervention or control group and a postnatal intervention or control group. The intervention group will receive daily BEP supplement to be consumed under supervision for the duration of pregnancy/lactation. Both intervention and control groups will receive the standard IFA tablet through the regular antenatal care program. In addition to the main trial, we propose a number of sub-studies to test specific hypotheses in a subsample of pregnant/lactating women and children. MISAME III started off with a formative research to identify the preferred product type for the provision of a fortified BEP supplement for the RCT.

Study setting

The study will be conducted in the district of Houndé in Burkina Faso, a land locked country situated in West-Africa. Burkina Faso has an infant mortality rate of 53 per 1.000 livebirths (17) with an estimated prevalence of LBW at 14% in 2013 (18). The prevalence of SGA has been estimated to be between 32.2% and 41.6% in the district of Houndé (19). The Demographic and Health Survey (DHS) of 2010 found that 16% of women had a body mass index (BMI) below 18.5 kg/m^2, which indicates that chronic energy deficiencies are present (20). The highest prevalence can be found in the Eastern region where 31% of women have a BMI lower than 18.5 kg/m^2 (21). And in particular, adolescent Burkinabe girls between the age of 15 and 19 years have a low BMI with a prevalence of 23% (22). Micronutrient deficiencies also still remain a problem in both infants and women of reproductive age in the country (22, 23).

The climate of the country is Sudano-Sahelian, with a dry season from October to March/April and a rainy season from May until September/October. The diet is essentially cereal-based (24) with maize as staple food (25).

For peer review only - http://bmjopen.bmj.com/site/about/guidelines.xhtml

MISAME III will be conducted in the same health district where two previous MISAME studies were organized (19, 26). The study villages are concentrated around six health centers which are within an accessible range from the district hospital. A list of all study sites can be found on *www.misame3.ugent*.

Study population and recruitment

Women living in the study villages and between the age of 15 and 40 years will be identified through a census. The villages have been selected based on their accessibility and distance to the nearest health centre, number of facility-based deliveries per year and their agricultural model as some households tend to reside on their fields during the harvest season. Trained village women (*femmes accompagnantes*: FA), selected in collaboration with the community leaders, will visit the households every five weeks to ask about women's menstruation. In case of amenorrhea, women will be sent to the nearest health center for a pregnancy test and a first antenatal consultation by our project midwife when tested positive. An ultrasound examination will be done soon after inclusion by the project medical doctor to assess gestational age. A baseline interview will also be done by the project interviewers to assess the household members characteristics, household properties, water sanitation and hygiene (WASH) and household food security.

Inclusion criteria:

- Women between 15-40 years old at study inclusion;
- Confirmed pregnancy by a pregnancy test;
- Women who signed the informed consent form.

Exclusion criteria:

- Pregnancies > 20 weeks of gestational age;
- Women planning on leaving the area during their pregnancy;
- Women planning on delivering outside the study area;
- Women who are allergic to peanuts;

FA will be informed by the project midwife when a participant has been included. FA will visit pregnant women on a daily basis to hand out the BEP supplement and/or IFA tablet and to supervise consumption. During the postnatal period, FA will hand out the supplements and IFA tablets to the intervention group on a daily basis until six weeks after birth. From that moment, they will receive a week-worth of BEP supplements. The postnatal control group will get the IFA tablets on a daily basis during the first six weeks after birth, and participants will be visited once a week afterwards (without any supplementation) to minimalize the effect of the home visits. The FA will inform women on the

For peer review only - http://bmjopen.bmj.com/site/about/guidelines.xhtml

supplement's function, the importance of antenatal visits during pregnancy, maintaining a healthy diversified diet, the importance of delivering at a health facility, the importance of exclusive breastfeeding and the introduction of complementary foods at the age of six months. Throughout the study, the FA will be supervised by project interviewers. Supervision visits will be conducted by using Lot Quality Assurance Sampling (LQAS) schemes and empty sachets counts to ensure that study participant are visited according to the project protocol.

Manufacturing of twelve fortified BEP supplements and the formative study

Twelve fortified BEP supplements were pre-tested before the start of the RCT during a formative research phase. Several food manufacturing companies were invited to produce ready-to-use BEP supplements following the compositional guidelines proposed during an expert meeting hosted by Bill and Melinda Gates Foundation (BMGF) (27) in September 2016 **(table 1)**. The BEP supplements had to be (i) ready to consume, (ii) not needing for a cold chain and (iii) microbiologically stable.

Table 1 *The compositional guidelines for macro- and micronutrients*

Nutrition component	Target per serving
Total energy:	250-500 kcal per serving;
Fat content:	10-60% of energy intake;
Protein content:	16 g (14-18 g) with a digestible indispensable amino acid score (DIAAS) of $\geq$ 0.9;
Carbohydrates:	between 45g and 32g per 100g (added saccharose between 20g and 10g per 100g);
Trans fats:	<1% of energy intake;
Fatty Acid	(optional): min of 1.3g of n-3 or 300mg docosahexaenoic acid (DHA) + eicosapentaenoic acid (EPA) (of which 200mg DHA) to achieve a healthy n-6: ratio of the supplement of 5:1;
Micronutrients:	vitamin A, D, E, K, B1 (thiamin), B2 (riboflavin), B3 (niacin), B6 (pyridoxine), B9 (folate), B12 and C; minerals: iron, zinc, iodine, calcium, phosphorous, copper, and selenium;
Optionally:	pantothenic acid, manganese, potassium, biotin and choline will be included;
	The final composition of the product will be determined by the selected product as the manufacturing process will influence the macronutrient composition.

Seven out of 12 were characterized as sweet and five as savoury. Products have been produced in different forms including a biscuit, pillow, wafer, bar, paste, instant drink, and soup

For peer review only - http://bmjopen.bmj.com/site/about/guidelines.xhtml

In a first screening step of the formative research, the two most preferred BEP were identified by using a combined evaluation approach consisting of a single meal test, sensory evaluation, and focus group discussions in a convenience sample of 40 pregnant women. In a next step, we compared the acceptability of the two pre-selected BEP supplements using a 10-week home-feeding study with 80 pregnant women to select to most preferred product for the RCT. We refer to both papers for detailed information (manuscripts under review).

Study intervention

At inclusion, pregnant women will be randomly allocated to four different study groups; (i) prenatal intervention only, (ii) postnatal intervention only, (iii) prenatal and postnatal intervention or (iv) no prenatal and no postnatal intervention. Prenatal and postnatal supplementation will start right after inclusion and birth, respectively. The prenatal intervention group will receive the fortified BEP and IFA, the control group IFA tablets alone. The IFA tablets contain 65mg iron and 0.4mg folic acid. The postnatal intervention group will receive the BEP supplement during six months together with the IFA tablets for six weeks following the national protocol of Burkina Faso, the control group will receive IFA alone for those six weeks.

Allocation/randomization

A stratified health center permuted block randomization schedule will be used to allocate women to the intervention and control group. The double random sequence will be generated before the start of the study using Stata 15.1 (Statacorp, Texas) by an external research analyst. A time coded Stata log-file is available upon request after the completion of the study to assess the randomization sequence generation. The allocation group will be coded with two letters (pre- and postnatal study group) and placed in a sequentially numbered sealed envelope by project employees not in direct contact with participants. The trial midwife will draw a sealed envelope at inclusion and allocate the participant to the group defined in the envelop. Blinding of participants and community-based project workers will not be possible since the supplements are identifiable. Blinding of outcome assessors will be ensured to a maximum extent measuring primary and secondary outcomes.

Outcomes

Primary outcomes of the RCT

The trial has two primary study outcomes that is used to assess the impact of the prenatal and the postnatal intervention respectively :

For peer review only - http://bmjopen.bmj.com/site/about/guidelines.xhtml

- Incidence of SGA , defined as birth weight <10th centile Intergrowth 21st reference (28);
- Length-for-age z-score (LAZ) calculated using the WHO 2006 growth reference at six months of age (29).

Secondary outcomes of the RCT

The trial has a list of secondary outcomes which can be found in table 2.
Birthweight measurements will be defined using the Intergrowth 21st reference (28) and child anthropometry using the Child Growth Standards developed by the WHO (29).

Table 2 *Secondary outcomes of the RCT on maternal, newborn and child level*

Maternal outcomes	Newborn	Child
Total and trimester specific prenatal weight gain and gestational weight change	Birth weight (measured within 72 hours after birth)	Weight-for-Age Z-score at six months of age (WAZ) (and 9 and 12 months on a subsample)
Probable and possible maternal post-natal depression at 2 and 6 months of child age	Birth length (measured within 72 hours after birth)	Weight-for-Length Z-score at six months of age (WLZ) (and 9 and 12 months on a subsample)
Maternal anemia at the third antenatal consultation	Ponderal or Rohrer's index at birth	Stunting at six months of age
Women's mean and minimum dietary diversity score (measured biweekly)	Gestational age at delivery	Wasting at six months of age
	Large-for-gestational age	Underweight at six months of age
	Chest circumference (measured within 72 hours after birth)	Duration of exclusive breastfeeding during the first six months of age
	Head circumference (measured within 72 hours after birth)	Incidence of child wasting over first six months of life
	Arm circumference (measured within 72 hours after birth)	Weight gain over first six months of life
	Incidence of preterm birth	Child mortality (between birth and six months of age)

For peer review only - http://bmjopen.bmj.com/site/about/guidelines.xhtml

	Fetal loss	Monthly change in LAZ over first six months of life
	Stillbirths	Monthly change in WHZ over first six months of life
		Monthly change in WAZ over first six months of life
		Monthly change in head circumference over first six months of life
		Child morbidity symptoms over first six months of life
		Anemia at six months of age
		Hemoglobin concentration at six months of age.

Outcomes of the sub studies

Sub-study 1: Impact of the intervention on child and maternal body composition.

To compare body composition between intervention and control, body composition will be determined in mother-child dyads by deuterium dilution, and analysis of saliva by a Fourier Transform Infrared reader (Agilent FTIR 4500 series).

Sub-study 2: Impact of the intervention on dietary intake.

A dietary assessment study will be conducted by using 24-hour recalls in a subsample of women. This will enable to determine if the food supplement actually supplements the diet or whether there is a degree of dietary substitution.

Sub-study 3: Impact of the intervention on breastmilk.

Breastmilk samples will be taken in the four study groups to compare the composition and to analyze the interaction between the supplementation periods.

Sample size

With an SGA prevalence of 32% and an anticipated decrease of 7%, a sample of 652 subjects per arm is required with $\alpha = 0.05$ and $\beta = 0.2$ (26). To accommodate for possible losses, the subjects per arm was increased to 888 (total subjects of 1776). Possible losses are based on previous MISAME studies

For peer review only - http://bmjopen.bmj.com/site/about/guidelines.xhtml

where the number was ~26 % due to a combination of abortions, still births, multifetal pregnancies, out-migrants, maternal deaths and incomplete data (19, 26). For the analysis of an effect of the postnatal intervention on length-for-age at six months of age, the minimally detectable effect depends on the presence or absence of an interaction effect between the pre- and postnatal intervention. In the absence of a statistically significant interaction between pre- and postnatal intervention, the sample size of 588 children per postnatal study arm allows to detect a difference of 0.18 Z-score (SD=1.1), based on a cross-sectional survey conducted in the Gourcy health district in Burkina Faso (30), between study arms with α=0.05 and β=0.20. This implies that if ~1400 singleton live births are available, we allow for a maximum lost to follow-up of 16%. In the presence of a statistically significant interaction between pre- and postnatal intervention, a total sample size of 1176 represents 294 children per factorial combination of the prenatal and postnatal study group (four groups in total). A subgroup size of 294 allows to detect a difference in LAZ at six months of child age of 0.28 assuming a SD of 1.1, α=0.025 (Bonferroni correction for two primary endpoints analyses) and β=0.20.

Data collection

Anthropometric and clinical procedures

At enrolment, anthropometric measurements from all women will be taken. Gestational age will be determined during an ultrasound consultation by measuring crown-rump length (7-13 weeks), or by calculating the mean of three to four measurements: biparietal diameter, head circumference, abdominal circumference, and femur length (12-26 weeks) (31).

During pregnancy, clinical follow up will consist of antenatal visits following the national guidelines.

At birth, anthropometric measurements of all neonates will be assessed in duplicate within the first 72 hours of life (in practice the aim is to measure within the 24 hours of life). After birth, mother and child will visit the health care centers monthly for a follow up on clinical, anthropometric and child morbidity measures (signs including fever, vomiting, diarrhea, cough, difficulty breathing and running nose). A subsample will be visited at home by the project interviewers to collect data at month 9 and 12.

Hemoglobin concentrations will be measured in women at enrolment and during the 3th antenatal care visit. This will be done at six months of age in children.

Baseline questionnaires

Pre- and postnatal maternal depression will be assessed using the standardized EPDS questionnaire consisting of 10 questions (32). Project midwives will be trained for this and the questionnaire will be

For peer review only - http://bmjopen.bmj.com/site/about/guidelines.xhtml

asked at inclusion and at month two and 6 after birth. Socio-economic and demographic information from all participants will be collected once included. Trained project interviewers will ask questions on household members characteristics, household properties, WASH environment and household food security (33). The women's dietary score will be measured in all participating women by the FA during the home visits. This will be done twice a week per participant using the Women's dietary diversity score with 11 food groups (34).

Table 3 shows the overview of the time schedule and measurements of the trial.

Quality of all study data will be insured by a thorough training of all field staff. Procedures to handle tools for data collection (questionnaires, anthropometric and clinical measurement material and laboratory procedures) will be practiced during a training and pre-tested on the field during a dry-run of ± three months. Anthropometry standardizations of the field staff will be repeated throughout the trial on a regular basis. All anthropometric measurements will be taken in duplicate, newborns will be measured within the 72 hours after birth (preferably within the 24 hours) and all weighing scales and HemoCue 201+ devices will regularly be controlled on preciseness to ensure data quality. A WhatsApp group will be set up where problems can be communicated and solved quickly.

All data collection forms of the trial can be found on *www.misame3.ugent.*

Women will be designated as lost to follow up if they move away or withdraw. Reasons for discontinuation will be recorded.

For peer review only

For peer review only - http://bmjopen.bmj.com/site/about/guidelines.xhtml

Table 3 *Participant timeline schedule of enrolment, interventions, assessment and visits.*

	**Enrolment**		**Allocation**	**Post-allocation**											
				**Pregnancy and birth**				**After birth**							
	**Start**	**5-weekly visits**	**ANC 1**	**Household visit**	**Ultra-sound**	**ANC 2, 3 and 4**	**Birth**	**Month 1**	**Month 2**	**Month 3**	**Month 4**	**Month 5**	**Month 6**	**Month 9**	**Month 12**
**Enrolment**															
Census	X														
Pregnancy identification		X													
Pregnancy confirmatory test			X												
Informed consent and allocation to study group			X												
**Study groups**															
Prenatal BEP + IFA			X	—	—	—	X								
Prenatal IFA			X	—	—	—	X								
Postnatal BEP + IFA*							X	—	—	—	—	—	X		
Postnatal IFA							X	—	X						

For peer review only - http://bmjopen.bmj.com/site/about/guidelines.xhtml

**Assessments**															
Mothers															
Baseline questionnaire				X											
Gestational age determination					X										
Skinfold measurements					X										
Weight (kg) and arm circumference (mm) **/***			X			X	X	X	X	X	X	X	X	X	X
Height (cm)			X												
Hemoglobin (g/dL)						X (ANC3)		X							
Women's dietary diversity score (biweekly)				X											
Maternal depression			X						X				X		
Infants															
Birth weight (kg)							X								
Birth length (cm)							X								
Head circumference (mm)							X	X	X	X	X	X	X		

For peer review only

Chest circumference (mm)							X								
Arm circumference (mm)								X		X			X		X
Morbidity								X	X	X	X	X	X		
Mortality							X	X	X	X	X	X	X		
Weight (kg) and height (cm)								X	X	X	X	X	X	X	X
Breastfeeding practices								X	X	X	X	X	X		
Hemoglobin (g/dL)													X		

* The IFA tablets will be given during the first 6 weeks after birth in the postnatal intervention group, according to the national health protocol.
** Only maternal weight will be taken at birth.
*** In subsample during month 9 and 12

For peer review only

For peer review only - http://bmjopen.bmj.com/site/about/guidelines.xhtml

Data management and analysis

FA will use smartphones with a CAPI programmed in CSPRO (version 7.3.1) to collect data during household visits. The study data collected by the project medical doctor, project midwives and interviewers will be done by Survey Solutions data entry software (version 19.12.6) on tablets. This data will be uploaded to a central server on a weekly basis. All questionnaires were programmed and have been tested on the Survey Solutions Designer website and include validation codes to promote the quality of the data entry in the field. Assignments will be sent once a week to the tablets of the field team and preloaded data collected at an earlier contact moment will be used to lower the amount of incorrect data. Paper forms will also be available on the field as a backup.

Further data quality checks will be done in Stata 15.1 (Statacorp, Texas). Missing or inconsistent data outliers will be sent back to the field for revision.

Statistical analysis

We refer to the Statistical Analysis Plan (SAP) of the trial; *"Statistical analysis plan: Impact of a prenatal and postnatal balanced energy protein supplement on birth size and postnatal child growth in Burkina Faso"* published at *www.misame3.ugent.*

Data monitoring

Data monitoring and auditing

The Data and Safety Monitoring Board (DSMB) is an independent multidisciplinary group whose members are not involved in the trial. The board consists of a Belgian endocrinologist, a Belgian pediatrician, a Burkinabe pediatrician, a Belgian gynecologist and a Belgian ethicist.

Serious adverse events (SAE)

FA will be trained to recognize health issues and will refer those participants actively to see the project midwife in the CSPS. All SAEs will be recorded on a case-by-case basis and verbal autopsies will be conducted for maternal, neonatal and infant deaths by the field medical doctor.

Patient and Public involvement

MISAME III has been well accepted by the community because of the previous positive experience they had with the MISAME I and II study. Through the formative research, women were involved in the

For peer review only - http://bmjopen.bmj.com/site/about/guidelines.xhtml

choice of BEP supplement. Workshops will be planned at in the end of the study in order to communicate the results to the community.

Ethics and dissemination

Ethics approval and consent to participate

MISAME III has been reviewed and approved by the University Hospital of the Ghent University and the Burkinabe ethics committee. Important protocol changes will be noted on *ClinicalTrials.gov*. When eligible women meet the inclusion criteria, project midwives will explain the background and procedure of the complete trial. Written informed consent or assent will be asked from the participating women. In case of illiteracy, a thumb print will be asked and witnessed by the recruiting investigator and one extra witness. Participants will be explained that all data collected during the trial is confidential and that they are allowed to withdraw at any time. A copy of the informed consent and assent can be found on *www.misame3.ugent*

Confidentiality

A data management plan has been put in place to address concerns regarding the General Data Protection Regulation (GDPR) rules. During the trial, the data files containing personal identifying information will be stored on the Survey Solutions server. Only the principal investigators and the project coordinators will be able to access those files.

Dissemination plan

Upon completion of the trial, all anonymized study data will be available upon request. Final results will be communicated to the participants, the Burkinabe Ministry of health, the field staff, the BMGF, the Ghent University researchers and students, AfricSanté, healthcare professionals and other relevant international public institutions. Papers on the study results will be published in peer-review journals and will be available on the project website. All investigators contributing to the realization of the project and publication of results will be an author. Other contributors such as the participants, FA and field staff members will be mentioned in the acknowledgments.

Ancillary care

The MISAME III project will pay for ancillary care when participants have health issues and in case the costs are not covered by the national health care program. Participants suffering harm from trial participation will be covered.

For peer review only - http://bmjopen.bmj.com/site/about/guidelines.xhtml

Discussion

In this paper the protocol of an individually randomized efficacy trial in rural Burkina Faso has been described in which pregnant and lactating women in the intervention group will receive a BEP supplement together with IFA tablets. The control group will only receive the standard IFA treatment.

The key features of the present trial are first, the inclusion of a formative research for a better understanding of which format of supplement is preferred, what taste is most acceptable, and which factors affect adherence in the study population. Second, the supplementation will not only be given during pregnancy but also during the first six months after birth. This will give the opportunity to assess the specific value of postnatal supplementation on several outcomes. Third, the observed daily intake of intervention and control supplements is a key feature to ensure compliance and to avoid sharing of the supplements with other household members. Fourth, MISAME III has the advantage of being the third trial of its kind in the study area. This presents an opportunity to anticipate the issues that arose in previous trials. For instance, women in specific villages tended to leave their homes for a longer period to go work on the fields outside the village. This gave problems in the continuation of the supplementation in the past and will be taken into consideration. And last, similar studies are being conducted in other countries, allowing the comparison of results from different contexts.

In addition, body composition will allow us to give insight in the mechanism by which prenatal supplementation affects birth anthropometry. These analyses will provide additional insights in the mechanisms by which the prenatal and postnatal interventions affect birth size and postnatal growth, respectively.

Overall, the MISAME III study is expected to improve the birth outcomes of neonates born from malnourished women in rural Burkina Faso. This will strengthen and refine the recommendation of the WHO concerning the use of context-specific BEP supplementation during pregnancy and lactation.

Supplementary file

SPIRIT Checklist 2013.

Abbreviations

AE; Adverse Events, ANC; Antenatal Care, BEP; Balanced Energy Supplement, BMGF; Bill and Melinda Gates Foundation, BMI; Body Mass Index, CSPro; Census and Survey Processing System, CSPS; Centre de Santé et de Promotion Social, DHS; Demographic and Health Survey, DSMB; Data and Safety Monitoring Board, EP(D)S; Edinburgh Postnatal (Postpartum) Depression Scale, FA; Femme

For peer review only - http://bmjopen.bmj.com/site/about/guidelines.xhtml

Accompagnante, GDPR; General Data Protection Regulation, HDI; Human Development Index, IFA; Iron/Folic Acid, IUGR; Intra Uterine Growth Restriction, LAZ; Length-for-Age Z-score, LBW; Low Birth Weight, LMIC; Low and Middle Income Countries, LMP; Last Menstrual Period, LQAS; Lot Quality Assessment Sampling, MISAME; MIcronutriments pour la SAnté de la Mère et de l'Enfant, MMS; Multiple-Micronutrient Supplements, RCT; Randomized Controlled Trial, SAE; Serious Adverse Events, SGA; Small-for-Gestational Age, UNICEF; United Nations International Children's Emergency Fund, UNIMMAP; UNICEF/WHO/UNU International Multiple Micronutrient, WASH; water, sanitation and hygiene, WAZ; Weight-for-Age Z-score, WHO; World Health Organization, WHZ; Weight-for-Height Z-score, WLZ; Weight-for-Length Z-score.

Acknowledgements

The MISAME Study Group thanks the pregnant women and their families for the time spent in this study. We would like to acknowledge the staff of AFRICSanté; including Henri Somé for his support on the CAPI software, the field medical doctor Anderson Compaoré, the data collectors (midwives, interviewers and femmes accompagnantes), dr. Alain Hein for the standardization exercises and dr. Hermann Lanou for the support during the training. We would like to thank dr. Sheila Isanaka for the collaboration during the formative research. Private sector partner Nutriset (France) is acknowledged for the development and production of the BEP supplements.

Competing interests statement

KV: none to declare
BdK: none to declare
LCT: none to declare
NDC: none to declare
MO: none to declare
RG: none to declare
CL: none to declare
LH: none to declare
PK: none to declare

Contributor Statement

The authors' responsibilities were as follows:
KV wrote the manuscript;
PK, LH, CL, NDC, LCT, KV, BdK designed the study and the protocol;
PK, LH, LCT, KV, BdK designed the study material tools;
LCT, LH, KV, BdK, MO, RG trained the field data collectors;
All authors contributed substantially to the manuscript and approved the final version.

Correspondence

Katrien.Vanslambrouck@ugent.be

Funding Information

This work was supported by the Bill and Melinda Gates Foundation. Grant number OPP1175213.

For peer review only - http://bmjopen.bmj.com/site/about/guidelines.xhtml

For peer review only

The trial is registered on clinical trials.gov (identifier: NCT00909974) prior to recruitment. Enrolment started in October 2019.

REFERENCES

1. Lee AC, Katz J, Blencowe H, Cousens S, Kozuki N, Vogel JP, et al. National and regional estimates of term and preterm babies born small for gestational age in 138 low-income and middle-income countries in 2010. The Lancet Global Health. 2013;1(1):e26-e36.
2. Katz J, Lee AC, Kozuki N, Lawn JE, Cousens S, Blencowe H, et al. Mortality risk in preterm and small-for-gestational-age infants in low-income and middle-income countries: a pooled country analysis. The Lancet. 2013;382(9890):417-25.
3. van Rossum CTM, Fransen HP, Verkaik-Kloosterman J, Buurma-Rethans EJM, Ocké MC. Dutch National Food Consumption Survey 2007–2010: Diet of children and adults aged 7 to 69 years2011.
4. Lee AC, Kozuki N, Cousens S, Stevens GA, Blencowe H, Silveira MF, et al. Estimates of burden and consequences of infants born small for gestational age in low and middle income countries with INTERGROWTH-21st standard: analysis of CHERG datasets. bmj. 2017;358:j3677.
5. Stevens B, Buettner P, Watt K, Clough A, Brimblecombe J, Judd J. The effect of balanced protein energy supplementation in undernourished pregnant women and child physical growth in low-and middle-income countries: a systematic review and meta-analysis. Maternal & child nutrition. 2015;11(4):415-32.
6. World Health Organization. WHO recommendations on antenatal care for a positive pregnancy experience: World Health Organization; 2016.
7. Krishna U, Bhalerao S. Placental insufficiency and fetal growth restriction. The Journal of Obstetrics and Gynecology of India. 2011;61(5):505-11.
8. Mridula D, Mishra C, Chakravorty A. Dietary intake of expectant mother. Indian J Nutr Diet. 2003;40(1):24-30.
9. Tang AM, Chung M, Dong K, Terrin N, Edmonds A, Assefa N, et al. Determining a global mid-upper arm circumference cutoff to assess malnutrition in pregnant women. Food and Nutrition Technical Assistance. 2016.
10. Branca F, Grummer-Strawn L, Borghi E, Blössner Md, Onis Md. Extension of the WHO maternal, infant and young child nutrition targets to 2030. SCN News. 2015(41):55-8.
11. Keats EC, Haider BA, Tam E, Bhutta ZA. Multiple-micronutrient supplementation for women during pregnancy. Cochrane Database of Systematic Reviews. 2019(3).
12. Hambidge KM, Westcott JE, Garcés A, Figueroa L, Goudar SS, Dhaded SM, et al. A multicountry randomized controlled trial of comprehensive maternal nutrition supplementation initiated before conception: the Women First trial. The American journal of clinical nutrition. 2019;109(2):457-69.
13. Ota E, Hori H, Mori R, Tobe-Gai R, Farrar D. Antenatal dietary education and supplementation to increase energy and protein intake. Cochrane Database of Systematic Reviews. 2015(6).
14. Imdad A, Bhutta ZA. Maternal nutrition and birth outcomes: Effect of balanced protein-energy supplementation. Paediatric and Perinatal Epidemiology. 2012;26:178-90.
15. Kramer MS, Kakuma R. Energy and protein intake in pregnancy. Cochrane database of systematic reviews. 2003(4).
16. Bill and Melinda Gates Foundation (BMGF). Framework and Specifications for the Nutritional Composition of a Food Supplement for Pregnant and Lactating Women (PLW) in Undernourished and Low-Income Settings. Expert consultation Seatle, US; 2016 2016.
17. United Nations Development Programme. Human Development Reports [Internet]. 2019 [cited 25 February 2020]. Available from: http://hdr.undp.org/en/countries/profiles/BFA.
18. Unicef. The state of the world's children: children with disabilities: UN; 2013.

For peer review only - http://bmjopen.bmj.com/site/about/guidelines.xhtml

19. Roberfroid D, Huybregts L, Lanou H, Henry M-C, Meda N, Menten J, et al. Effects of maternal multiple micronutrient supplementation on fetal growth: a double-blind randomized controlled trial in rural Burkina Faso. The American journal of clinical nutrition. 2008;88(5):1330-40.
20. INSD MD, Macro I. Enquête démographique et de santé et à indicateurs multiples (EDSBF-MICS IV), Rapport préliminaire, Burkina Faso, 2010. Ouagadougou: INSD. 2011;40.
21. Institut National de la Statistique et de la Démographie (INSD) et ICF International. Enquête Démographique et de Santé et à Indicateurs Multiples du Burkina Faso 2010. Calverton, Maryland, USA : INSD et ICF International. 2012.
22. MOH. National Iodine Status and Anemia Survey, Burkina Faso. 2014.
23. MOH. MdlS. Enquête Nutritionnelle Nationale 2013. 2013.
24. Savy M, Martin-Prével Y, Sawadogo P, Kameli Y, Delpeuch F. Use of variety/diversity scores for diet quality measurement: relation with nutritional status of women in a rural area in Burkina Faso. European journal of clinical nutrition. 2005;59(5):703.
25. Huybregts LF, Roberfroid DA, Kolsteren PW, Van Camp JH. Dietary behaviour, food and nutrient intake of pregnant women in a rural community in Burkina Faso. Maternal & child nutrition. 2009;5(3):211-22.
26. Huybregts L, Roberfroid D, Lanou H, Menten J, Meda N, Van Camp J, et al. Prenatal food supplementation fortified with multiple micronutrients increases birth length: a randomized controlled trial in rural Burkina Faso. Am J Clin Nutr. 2009;90(6):1593-600.
27. Cuzzocrea F, Larcan R, Lanzarone C. Gender differences, personality and eating behaviors in non-clinical adolescents. Eat Weight Disord-St. 2012;17(4):E282-E9.
28. Villar J, Altman D, Purwar M, Noble J, Knight H, Ruyan P, et al. The objectives, design and implementation of the INTERGROWTH-21st Project. BJOG: An International Journal of Obstetrics & Gynaecology. 2013;120:9-26.
29. De Onis M, Garza C, Onyango AW, Martorell R. WHO child growth standards: Taylor & Francis Philadelphia, PA; 2006.
30. Becquey E, Huybregts L, Zongrone A, Le Port A, Leroy JL, Rawat R, et al. Impact on child acute malnutrition of integrating a preventive nutrition package into facility-based screening for acute malnutrition during well-baby consultation: A cluster-randomized controlled trial in Burkina Faso. PLoS medicine. 2019;16(8).
31. Salomon L, Alfirevic Z, Da Silva Costa F, Deter R, Figueras F, Ghi T, et al. ISUOG Practice Guidelines: ultrasound assessment of fetal biometry and growth. Ultrasound in Obstetrics & Gynecology. 2019;53(6):715-23.
32. Cox JL, Holden JM, Sagovsky R. Detection of postnatal depression: development of the 10-item Edinburgh Postnatal Depression Scale. The British journal of psychiatry. 1987;150(6):782-6.
33. Femi A. Perception of performance appraisal and workers' performance in Wema Bank Headquarters, Lagos. Global Journal of Arts, Humanities and Social Sciences. 2013;1(4):89-101.
34. Martin-Prével Y, Allemand P, Wiesmann D, Arimond M, Ballard T, Deitchler M, et al. Moving forward on choosing a standard operational indicator of women's dietary diversity. FAO; 2015.

For peer review only - http://bmjopen.bmj.com/site/about/guidelines.xhtml

Figure 1 *Study design of the RCT*

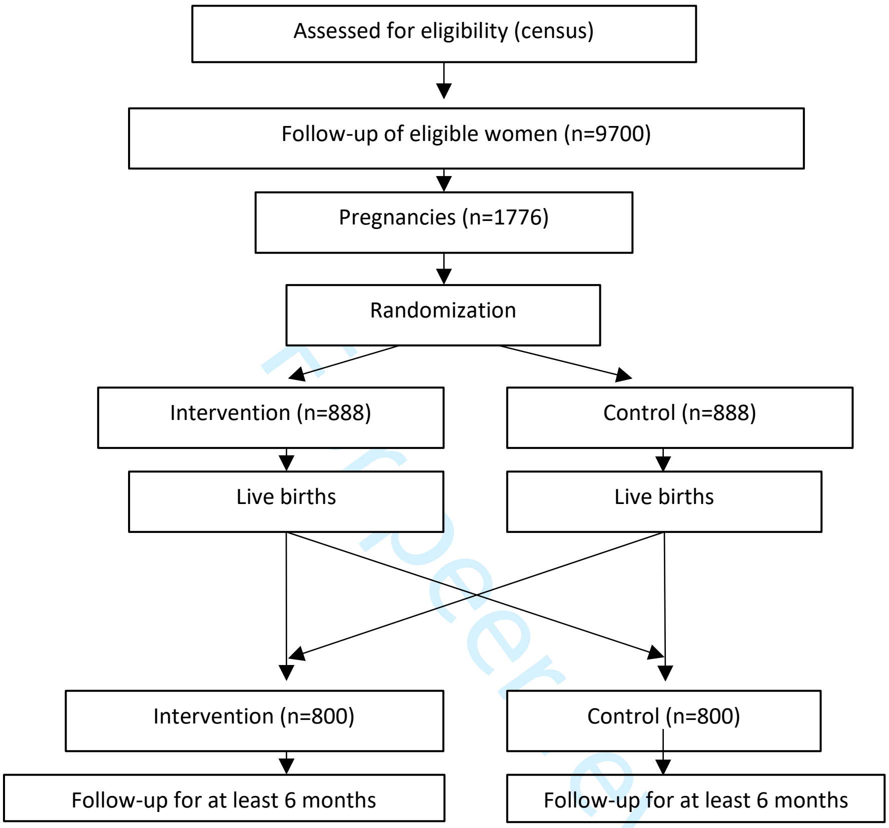

For peer review only

Supplementary file : SPIRIT Checklist 2013.

Reporting checklist for protocol of a clinical trial.

Based on the SPIRIT guidelines.

Instructions to authors

Complete this checklist by entering the page numbers from your manuscript where readers will find each of the items listed below.

Your article may not currently address all the items on the checklist. Please modify your text to include the missing information. If you are certain that an item does not apply, please write "n/a" and provide a short explanation.

Upload your completed checklist as an extra file when you submit to a journal.

In your methods section, say that you used the SPIRITreporting guidelines, and cite them as:

Chan A-W, Tetzlaff JM, Altman DG, Laupacis A, Gøtzsche PC, Krleža-Jerić K, Hróbjartsson A, Mann H, Dickersin K, Berlin J, Doré C, Parulekar W, Summerskill W, Groves T, Schulz K, Sox H, Rockhold FW, Rennie D, Moher D. SPIRIT 2013 Statement: Defining standard protocol items for clinical trials. Ann Intern Med. 2013;158(3):200-207

		Reporting Item	Page Number
**Administrative information**			
Title	#1	Descriptive title identifying the study design, population, interventions, and, if applicable, trial acronym	1
Trial registration	#2a	Trial identifier and registry name. If not yet registered, name of intended registry	2
Trial registration: data set	#2b	All items from the World Health Organization Trial Registration Data Set	n/a
Protocol version	#3	Date and version identifier	1
Funding	#4	Sources and types of financial, material, and other support	20
Roles and responsibilities: contributorship	#5a	Names, affiliations, and roles of protocol contributors	1 / 20
Roles and responsibilities: sponsor contact information	#5b	Name and contact information for the trial sponsor	20

For peer review only

Roles and responsibilities: sponsor and funder	#5c	Role of study sponsor and funders, if any, in study design; collection, management, analysis, and interpretation of data; writing of the report; and the decision to submit the report for publication, including whether they will have ultimate authority over any of these activities	20
Roles and responsibilities: committees	#5d	Composition, roles, and responsibilities of the coordinating centre, steering committee, endpoint adjudication committee, data management team, and other individuals or groups overseeing the trial, if applicable (see Item 21a for data monitoring committee)	17
**Introduction**			
Background and rationale	#6a	Description of research question and justification for undertaking the trial, including summary of relevant studies (published and unpublished) examining benefits and harms for each intervention	4
Background and rationale: choice of comparators	#6b	Explanation for choice of comparators	4
Objectives	#7	Specific objectives or hypotheses	4
Trial design	#8	Description of trial design including type of trial (eg, parallel group, crossover, factorial, single group), allocation ratio, and framework (eg, superiority, equivalence, non-inferiority, exploratory)	5
**Methods: Participants, interventions, and outcomes**			
Study setting	#9	Description of study settings (eg, community clinic, academic hospital) and list of countries where data will be collected. Reference to where list of study sites can be obtained	6
Eligibility criteria	#10	Inclusion and exclusion criteria for participants. If applicable, eligibility criteria for study centres and individuals who will perform the interventions (eg, surgeons, psychotherapists)	6
Interventions: description	#11a	Interventions for each group with sufficient detail to allow replication, including how and when they will be administered	8

For peer review only

Interventions: modifications	#11b	Criteria for discontinuing or modifying allocated interventions for a given trial participant (eg, drug dose change in response to harms, participant request, or improving / worsening disease)	n/a
Interventions: adherance	#11c	Strategies to improve adherence to intervention protocols, and any procedures for monitoring adherence (eg, drug tablet return; laboratory tests)	7 /
Interventions: concomitant care	#11d	Relevant concomitant care and interventions that are permitted or prohibited during the trial	n/a
Outcomes	#12	Primary, secondary, and other outcomes, including the specific measurement variable (eg, systolic blood pressure), analysis metric (eg, change from baseline, final value, time to event), method of aggregation (eg, median, proportion), and time point for each outcome. Explanation of the clinical relevance of chosen efficacy and harm outcomes is strongly recommended	9
Participant timeline	#13	Time schedule of enrolment, interventions (including any run-ins and washouts), assessments, and visits for participants. A schematic diagram is highly recommended (see Figure)	14
Sample size	#14	Estimated number of participants needed to achieve study objectives and how it was determined, including clinical and statistical assumptions supporting any sample size calculations	11
Recruitment	#15	Strategies for achieving adequate participant enrolment to reach target sample size	6
**Methods: Assignment of interventions (for controlled trials)**			
Allocation: sequence generation	#16a	Method of generating the allocation sequence (eg, computer-generated random numbers), and list of any factors for stratification. To reduce predictability of a random sequence, details of any planned restriction (eg, blocking) should be provided in a separate document that is unavailable to those who enrol participants or assign interventions	8
Allocation concealment mechanism	#16b	Mechanism of implementing the allocation sequence (eg, central telephone; sequentially numbered, opaque, sealed envelopes), describing any steps to conceal the sequence until interventions are assigned	8

For peer review only

Allocation: implementation	#16c	Who will generate the allocation sequence, who will enrol participants, and who will assign participants to interventions	8
Blinding (masking)	#17a	Who will be blinded after assignment to interventions (eg, trial participants, care providers, outcome assessors, data analysts), and how	9
Blinding (masking): emergency unblinding	#17b	If blinded, circumstances under which unblinding is permissible, and procedure for revealing a participant’s allocated intervention during the trial	9
**Methods: Data collection, management, and analysis**			
Data collection plan	#18a	Plans for assessment and collection of outcome, baseline, and other trial data, including any related processes to promote data quality (eg, duplicate measurements, training of assessors) and a description of study instruments (eg, questionnaires, laboratory tests) along with their reliability and validity, if known. Reference to where data collection forms can be found, if not in the protocol	12
Data collection plan: retention	#18b	Plans to promote participant retention and complete follow-up, including list of any outcome data to be collected for participants who discontinue or deviate from intervention protocols	13
Data management	#19	Plans for data entry, coding, security, and storage, including any related processes to promote data quality (eg, double data entry; range checks for data values). Reference to where details of data management procedures can be found, if not in the protocol	17
Statistics: outcomes	#20a	Statistical methods for analysing primary and secondary outcomes. Reference to where other details of the statistical analysis plan can be found, if not in the protocol	17
Statistics: additional analyses	#20b	Methods for any additional analyses (eg, subgroup and adjusted analyses)	n/a
Statistics: analysis population and missing data	#20c	Definition of analysis population relating to protocol non-adherence (eg, as randomised analysis), and any statistical methods to handle missing data (eg, multiple imputation)	17
**Methods: Monitoring**			

For peer review only

Data monitoring: formal committee	#21a	Composition of data monitoring committee (DMC); summary of its role and reporting structure; statement of whether it is independent from the sponsor and competing interests; and reference to where further details about its charter can be found, if not in the protocol. Alternatively, an explanation of why a DMC is not needed	17
Data monitoring: interim analysis	#21b	Description of any interim analyses and stopping guidelines, including who will have access to these interim results and make the final decision to terminate the trial	n/a
Harms	#22	Plans for collecting, assessing, reporting, and managing solicited and spontaneously reported adverse events and other unintended effects of trial interventions or trial conduct	17
Auditing	#23	Frequency and procedures for auditing trial conduct, if any, and whether the process will be independent from investigators and the sponsor	n/a
**Ethics and dissemination**			
Research ethics approval	#24	Plans for seeking research ethics committee / institutional review board (REC / IRB) approval	18
Protocol amendments	#25	Plans for communicating important protocol modifications (eg, changes to eligibility criteria, outcomes, analyses) to relevant parties (eg, investigators, REC / IRBs, trial participants, trial registries, journals, regulators)	18
Consent or assent	#26a	Who will obtain informed consent or assent from potential trial participants or authorised surrogates, and how (see Item 32)	18
Consent or assent: ancillary studies	#26b	Additional consent provisions for collection and use of participant data and biological specimens in ancillary studies, if applicable	n/a
Confidentiality	#27	How personal information about potential and enrolled participants will be collected, shared, and maintained in order to protect confidentiality before, during, and after the trial	18
Declaration of interests	#28	Financial and other competing interests for principal investigators for the overall trial and each study site	20

For peer review only

Data access	#29	Statement of who will have access to the final trial dataset, and disclosure of contractual agreements that limit such access for investigators	18
Ancillary and post trial care	#30	Provisions, if any, for ancillary and post-trial care, and for compensation to those who suffer harm from trial participation	18
Dissemination policy: trial results	#31a	Plans for investigators and sponsor to communicate trial results to participants, healthcare professionals, the public, and other relevant groups (eg, via publication, reporting in results databases, or other data sharing arrangements), including any publication restrictions	18
Dissemination policy: authorship	#31b	Authorship eligibility guidelines and any intended use of professional writers	18
Dissemination policy: reproducible research	#31c	Plans, if any, for granting public access to the full protocol, participant-level dataset, and statistical code	n/a
**Appendices**			
Informed consent materials	#32	Model consent form and other related documentation given to participants and authorised surrogates	18
Biological specimens	#33	Plans for collection, laboratory evaluation, and storage of biological specimens for genetic or molecular analysis in the current trial and for future use in ancillary studies, if applicable	n/a

The SPIRIT checklist is distributed under the terms of the Creative Commons Attribution License CC-BY-ND 3.0. This checklist was completed on 06. March 2020 using https://www.goodreports.org/, a tool made by the EQUATOR Network in collaboration with Penelope.ai

For peer review only

BMJ Open

THE EFFECT OF BALANCED ENERGY-PROTEIN SUPPLEMENTATION DURING PREGNANCY AND LACTATION ON BIRTH OUTCOMES AND INFANT GROWTH IN RURAL BURKINA FASO : STUDY PROTOCOL FOR A RANDOMIZED CONTROLLED TRIAL

Journal:	*BMJ Open*
Manuscript ID	bmjopen-2020-038393.R1
Article Type:	Protocol
Date Submitted by the Author:	06-Jan-2021
Complete List of Authors:	Vanslambrouck, Katrien; University of Ghent, Food Technology, Safety and Health, Faculty of Bioscience Engineering<br>de Kok, Brenda; Ghent University, Department of Food Technology, Safety and Health, Faculty of Bioscience Engineering<br>Toe, Laeticia Celine; Ghent University, Department of Food Technology, Safety and Health, Faculty of Bioscience Engineering; Institut de Recherche en Sciences de la Sante<br>De Cock, Nathalie; Ghent University, Department of Food Technology, Safety and Health, Faculty of Bioscience Engineering<br>Ouedraogo, Moctar; AfricSanté<br>Dailey-Chwalibog, Trenton; Ghent University, Department of Food Technology, Safety and Health, Faculty of Bioscience Engineering<br>Hanley Cook, Giles; Ghent University, Department of Food Technology, Safety and Health, Faculty of Bioscience Engineering<br>Ganaba, Rasmané; AfricSanté<br>Lachat, Carl; Ghent University, Department of Food Technology, Safety and Health, Faculty of Bioscience Engineering<br>Huybregts, Lieven; IFPRI, PHN division<br>Kolsteren, Patrick; Ghent University, Department of Food Technology, Safety and Health, Faculty of Bioscience Engineering
**Primary Subject Heading**:	Nutrition and metabolism
Secondary Subject Heading:	Paediatrics
Keywords:	Nutrition < TROPICAL MEDICINE, Community child health < PAEDIATRICS, Protocols & guidelines < HEALTH SERVICES ADMINISTRATION & MANAGEMENT

SCHOLARONE™ Manuscripts

For peer review only - http://bmjopen.bmj.com/site/about/guidelines.xhtml

I, the Submitting Author has the right to grant and does grant on behalf of all authors of the Work (as defined in the below author licence), an exclusive licence and/or a non-exclusive licence for contributions from authors who are: i) UK Crown employees; ii) where BMJ has agreed a CC-BY licence shall apply, and/or iii) in accordance with the terms applicable for US Federal Government officers or employees acting as part of their official duties; on a worldwide, perpetual, irrevocable, royalty-free basis to BMJ Publishing Group Ltd ("BMJ") its licensees and where the relevant Journal is co-owned by BMJ to the co-owners of the Journal, to publish the Work in this journal and any other BMJ products and to exploit all rights, as set out in our licence.

The Submitting Author accepts and understands that any supply made under these terms is made by BMJ to the Submitting Author unless you are acting as an employee on behalf of your employer or a postgraduate student of an affiliated institution which is paying any applicable article publishing charge ("APC") for Open Access articles. Where the Submitting Author wishes to make the Work available on an Open Access basis (and intends to pay the relevant APC), the terms of reuse of such Open Access shall be governed by a Creative Commons licence – details of these licences and which Creative Commons licence will apply to this Work are set out in our licence referred to above.

Other than as permitted in any relevant BMJ Author's Self Archiving Policies, I confirm this Work has not been accepted for publication elsewhere, is not being considered for publication elsewhere and does not duplicate material already published. I confirm all authors consent to publication of this Work and authorise the granting of this licence.

For peer review only

[Title Page File]

Title:

THE EFFECT OF BALANCED ENERGY-PROTEIN SUPPLEMENTATION DURING PREGNANCY AND LACTATION ON BIRTH OUTCOMES AND INFANT GROWTH IN RURAL BURKINA FASO : STUDY PROTOCOL FOR A RANDOMIZED CONTROLLED TRIAL

Authors:

Vanslambrouck Katrien [a], de Kok Brenda [a], Toe Laeticia Celine [a c], De Cock Nathalie [a], Ouedraogo Moctar [d], Dailey-Chwalibog Trenton [a], Hanley-Cook Giles [a], Ganaba Rasmané [d], Lachat Carl [a], Huybregts Lieven [b], Kolsteren Patrick [a]

Authors institutional affiliations:

[a] Department of Food Technology, Safety and Health, Faculty of Bioscience Engineering, Ghent University, Ghent, Belgium

[b] Poverty, Health and Nutrition Division, International Food Policy Research Institute (IFPRI), 1201 I Street NW, Washington, DC 20006, USA

[c] Institut de Recherche en Sciences de la Santé (IRSS), Bobo-Dioulasso, Burkina Faso

[d] Africsanté, Bobo-Dioulasso, Burkina Faso

Word count :

4.000 words

Correspondence :

Katrien.Vanslambrouck@ugent.be - katvsb@hotmail.com

Faculty of Bioscience Engineering

Department of Food technology, Safety and Health

Coupure Links 653, geb. A, 9000 Gent

09/264 61 77

Other email addresses :

Brenda.deKok@UGent.be - LaeticiaCeline.Toe@UGent.be - nadecock@gmail.com

obmoctar@gmail.com – trenton.daileychwalibog@UGent.be – Giles.HanleyCook@UGent.be - rganaba@hotmail.com - Carl.Lachat@UGent.be – L.Huybregts@cgiar.org - Patrick.Kolsteren@UGent.be

For peer review only

For peer review only - http://bmjopen.bmj.com/site/about/guidelines.xhtml

[Main Text File]

Abstract

Introduction:

Adequate nutrition during pregnancy is crucial to both mother and child. Maternal malnutrition can be the cause of stillbirth or lead to poor birth outcomes such as preterm delivery and small-for-gestational-age new-borns. There is a probable positive effect of providing pregnant women a balanced energy-protein (BEP) food supplement but more evidence is needed. The MISAME III project aims to improve birth outcomes and infant growth by testing a BEP supplement during pregnancy and lactation in rural Burkina Faso. This paper describes the study protocol.

Methods and analysis:

MISAME III is a 4-arm individually randomized efficacy trial implemented in six rural health center catchments areas in the district of Houndé. Eligible pregnant women, aged between 15 and 40 years old and living in the study areas, will be enrolled. Women will be randomly assigned to one of the four study groups: (i) prenatal intervention only, (ii) postnatal intervention only, (iii) prenatal and postnatal intervention or (iv) no prenatal or postnatal intervention. The intervention group will receive the BEP supplement and iron/folic acid (IFA) tablets while the control group will only receive the IFA tablets following the national health protocol. Consumption will be supervised by trained village women on a daily basis by means of home visits. The primary outcomes are small-for-gestational age at birth and length-for-age z-score at six months of age. Secondary outcomes will be measured at birth and during the first six months of the infants' life. Women will be enrolled from October 2019 until the total sample size is reached.

Ethics and dissemination:

MISAME-III has been reviewed and approved by the University Hospital of Ghent and the ethics committee of Centre Muraz, Burkina Faso. Informed consent will be obtained. Results will be published in relevant journals and shared with other researchers and public health institutions.

Trial registration number on Clinical-Trials.gov: NCT03533712.

Protocol v2

15/12/2020

For peer review only - http://bmjopen.bmj.com/site/about/guidelines.xhtml

Strengths and limitations of this study

- This trial will help to fill the evidence gap on the effect of BEP supplements in pregnant and lactating women on birth outcomes and infant growth.
- Formative research to select the most suitable BEP supplement ensured that the selected BEP is well-accepted by the study population.
- The daily intake of BEP supplements and IFA tablets during pregnancy and lactation will be directly observed by study workers.
- This study will assess the impact of factorial combinations of pre-and postnatal BEP on child growth to elucidate the relative importance of BEP during pregnancy and/or early lactation.
- Blinding of study participants and staff members will not be possible, as the supplements are identifiable.

Keywords: Maternal nutrition, Dietary supplements, Pregnancy outcomes, Child growth, Small for Gestational Age, Length for Age, Burkina Faso

For peer review only - http://bmjopen.bmj.com/site/about/guidelines.xhtml

Introduction

Pregnancy is a challenging period in the life of many women in low- and middle-income countries (LMICs). Maternal mortality remains high and many neonates suffer from premature delivery and/or intrauterine growth retardation, both in length and in weight accumulation (1). An indicator to measure neonatal growth is small-for-gestational age (SGA). SGA is defined as a birthweight below the 10th percentile of a standard optimal reference population for a given gestational age and sex (2). SGA is often caused by growth-restriction in the womb and has been associated with neonatal and post-neonatal mortality (2). It has also been linked to an increased risk of morbidity later in life, especially non-communicable diseases (3). SGA affected 23.3 million term children in LMICs in 2012 (4). Adequate nutrition during pregnancy is crucial for optimal maternal and newborn health (5, 6) and maternal malnutrition has been associated with fetal growth restriction (7). An adequate dietary balance is necessary to ensure sufficient energy intake for adequate growth of the fetus (8). Unfortunately, maternal undernutrition remains a public health challenge in regions across sub-Saharan Africa and Asia (9, 10).

Several types of food supplements have been developed and evaluated over the past years. A positive effect of multiple-micronutrient supplements (MMS) during pregnancy on birth outcomes has been found in previous studies (11). Keats et al. concluded in their review that MMS during pregnancy gave a probable reduction in SGA and preterm births and can thus be used for future guidance (11). According to a multi-country randomized controlled trial (RCT) done in LMICs, a positive effect of lipid-based nutrient supplements on fetal growth-related birth outcomes can be seen when starting supplementation before conception or during the first trimester (12). Moreover, the latest evidence indicates a possible positive effect of providing pregnant women a balanced energy-protein (BEP) food supplement (5) (13) (14) (15). In line with that evidence, the 2016 World Health Organization's (WHO) antenatal care guidelines state that pregnant women in undernourished populations should receive, depending on the context, BEP supplements to reduce the risk of stillbirth and SGA (6). Researchers, however, still highlight the limited amount of evidence and a need to evaluate the effect of balanced supplements on birth outcomes, such as SGA (5, 13). Experimental trials of high quality and large sample sizes, especially in undernourished pregnant women, are thus needed (13). Following this recommendation, compositional guidance for a ready-to-use food supplement for pregnant women was developed by the Bill and Melinda Gates Foundation (BMGF) in 2016 (16).

For peer review only - http://bmjopen.bmj.com/site/about/guidelines.xhtml

Two previous projects, MISAME-I & II (MIcronutriments pour la SAnté de la Mère et de l'Enfant), conducted in Burkina Faso, investigated the effect of supplementation during pregnancy and the effect on birth outcomes in infants (17) (18). MISAME-I compared the effect of the UNICEF/WHO/UNU international multiple micronutrient preparation (UNIMMAP) with the effect of iron and folic acid (IFA) alone on fetal growth in a double-blind RCT and concluded that UNIMMAP modestly, but significantly increased fetal growth (17). The second study (MISAME-II) assessed the effect of a lipid-based nutrient supplement fortified with UNIMMAP compared to a UNIMMAP tablet during pregnancy in an open-label, individually randomized controlled trial on birth anthropometry. It was found that combining energy with micronutrients during the prenatal phase led to larger birth lengths (18). Since MISAME-II used the UNIMMAP as a control, a complete assessment of the impact of the fortified lipid-based UNIMMAP was not possible. Therefore, MISAME-III will study the effect of a BEP supplementation, compared to a control group, and extend the supplementation postnatally to investigate the net contribution of pre-and postnatal BEP supplementation on child linear growth up to 6 months of age.

In summary, the MISAME-III study hypothesizes that: (i) providing women with a BEP supplement during pregnancy will decrease the incidence of SGA compared to the control group; and, (ii) providing them with a BEP supplement during the postnatal period will increase children's length by the age of six months compared to the control group.

Methods

This protocol has been developed in accordance with the SPIRIT guidelines (**Supplementary file 1**).

MISAME III study design

The MISAME-III project is an individually randomized 2×2 factorial efficacy trial aiming to improve birth outcomes and infant growth in rural Burkina Faso by testing a BEP supplement during pregnancy and lactation **(Figure 1**). At inclusion, pregnant women will be individually and randomly allocated to a prenatal intervention or control group and a postnatal intervention or control group. The intervention group will receive a daily BEP supplement to be consumed under supervision for the duration of pregnancy/lactation. Both intervention and control groups will receive the standard IFA tablet through the national antenatal care In addition to the main trial, we propose a number of sub-studies to test specific hypotheses in a subsample of pregnant/lactating women and children. MISAME-III began with a formative study to identify the preferred product type for the provision of a fortified BEP supplement during the RCT.

Study setting

For peer review only

The study will be conducted in the district of Houndé in Burkina Faso, a land locked country situated in West-Africa, similar to the previous MISAME studies Burkina Faso has an infant mortality rate of 53 per 1.000 live births (19), with an estimated LBW prevalence at 14% in 2013 (20). The prevalence of SGA has been estimated to be between 32.2% and 41.6% in the district of Houndé (17). The Demographic and Health Survey (DHS) of 2010 reported that 16% of women had a body mass index (BMI) below 18.5 kg/m^2, which indicates the presence of chronic energy deficiencies in the zone(21). The highest prevalence can be found in the Eastern region, where 31% of women have a BMI lower than 18.5 kg/m^2 i.e., low BMI (22). Moreover, in particular, adolescent Burkinabèe girls between the age of 15 and 19 years have a low BMI, with an estimated prevalence of 23% (23). Micronutrient deficiencies also remain a major problem in both infants and women of reproductive age in the country (23, 24).

The climate of the country is Sudano-Sahelian, with a dry season from October to March/April and a rainy season from May until September/October. The diet is essentially cereal-based (25) with maize as the main staple food (26).

MISAME-III will be conducted in the same health district where the two previous MISAME studies were organized. The study villages are concentrated around six health centers, which are within an accessible range from the district hospital. A list of all study sites can be found on:*www.misame3.ugent*.

Study population and recruitment

Women living in the study villages and aged between 15 and 40 years will be identified through a census. The villages were selected based on their accessibility and distance to the nearest health centre, number of facility-based deliveries per year, and their agricultural model as some households tend to reside on their fields during the harvest season. Trained village women (*femme accompagnante*: FA), selected in collaboration with the community leaders, will visit the households every five weeks to ask about women's menstruation. In case of amenorrhea, women will be sent to the nearest health center for a pregnancy test and a first antenatal consultation by our project midwife when tested positive. An ultrasound examination will be completed soon after inclusion by the project medical doctor to assess gestational age. A baseline interview will also be done by the project interviewers to assess the household members' characteristics, household properties, water sanitation and hygiene (WASH) and household food security.

Inclusion criteria:

- Women between 15-40 years old at study inclusion;

For peer review only - http://bmjopen.bmj.com/site/about/guidelines.xhtml

- Confirmed pregnancy by a pregnancy test;
- Women who signed the informed consent form.

Exclusion criteria:

- Pregnancies > 20 weeks of gestational age;
- Women planning on leaving the area during their pregnancy;
- Women planning on delivering outside the study area;
- Women who are allergic to peanuts.

FAs will be informed by the project midwife when a participant has been included. FAs will visit pregnant women on a daily basis to distribute the BEP supplement and/or IFA tablet and to supervise consumption. During the postnatal period, FAs will distribute the supplements and IFA tablets to the intervention group on a daily basis until six weeks after birth. From that moment onwards, they will receive a week's worth of BEP supplements. The postnatal control group will receive the IFA tablets on a daily basis during the first six weeks after birth, and participants will thereafter be visited once a week (without any supplementation) to minimize the effect of home visits. The FA will inform women on the supplement's function, the importance of antenatal visits during pregnancy, maintaining a healthy diversified diet, the importance of delivering at a health facility, the importance of exclusive breastfeeding, and the introduction of complementary foods at the age of six months. Throughout the study, the FAs will be supervised by project interviewers. Supervision visits will be conducted using Lot Quality Assurance Sampling (LQAS) schemes and empty sachets counts to ensure that study participant are visited according to the project protocol.

Manufacturing of twelve fortified BEP supplements and the formative study

Twelve fortified BEP supplements were pre-tested before the start of the RCT during a formative research phase. Several food manufacturing companies were invited to produce ready-to-use BEP supplements following the compositional guidelines proposed during an expert meeting hosted by the Bill and Melinda Gates Foundation (BMGF) (16) in September 2016 **(Table 1)**. The BEP supplements had to be (i) ready to consume, (ii) not need for a cold chain, and (iii) microbiologically stable.

Table 1 *The compositional guidelines for macro- and micronutrients*

Nutrition component	Target per serving
Total energy:	250-500 kcal per serving;
Fat content:	10-60% of energy intake;
Protein content:	16 g (14-18 g) with a digestible indispensable amino acid score (DIAAS) of $\geq$ 0.9;

For peer review only - http://bmjopen.bmj.com/site/about/guidelines.xhtml

Carbohydrates:	between 45g and 32g per 100g (added sucrose between 20g and 10g per 100g);
Trans fats:	<1% of energy intake;
Fatty Acid	(optional): min of 1.3g of n-3 or 300mg docosahexaenoic acid (DHA) + eicosapentaenoic acid (EPA) (of which 200mg DHA) to achieve a healthy n-6: ratio of the supplement of 5:1;
Micronutrients:	vitamin A, D, E, K, B1 (thiamin), B2 (riboflavin), B3 (niacin), B6 (pyridoxine), B9 (folate), B12 and C; minerals: iron, zinc, iodine, calcium, phosphorous, copper, and selenium;
Optionally:	pantothenic acid, manganese, potassium, biotin and choline will be included;
	The final composition of the product will be determined by the selected product as the manufacturing process will influence the macronutrient composition.

Seven out of 12 supplements were characterized as sweet and five as savoury. Products were produced in different forms, including a biscuit, pillow, wafer, bar, paste, instant drink, and soup.

In a first screening step of the formative study, the two most preferred BEP supplements were identified by using a combined evaluation approach consisting of a single meal test, sensory evaluation, and focus group discussions, in a convenience sample of 40 pregnant women. In a next step, we compared the acceptability of the two pre-selected BEP supplements using a 10-week home-feeding study, with 80 pregnant women, to select to most preferred product for the RCT. We refer to both papers for detailed information (27) (28).

Study intervention

At inclusion, pregnant women will be randomly allocated to four different study groups; (i) prenatal intervention only, (ii) postnatal intervention only, (iii) prenatal and postnatal intervention or (iv) no prenatal and no postnatal intervention. Prenatal and postnatal supplementation will start right after inclusion and birth, respectively. The prenatal intervention group will receive the BEP supplement and IFA, the control group IFA tablets alone. The IFA tablets contain 65mg iron and 0.4mg folic acid. The postnatal intervention group will receive the BEP supplement for six months in combination with the IFA tablets for six weeks following the national protocol of Burkina Faso; the control group will receive IFA alone for six weeks.

Allocation/randomization

We will apply a stratified permuted block randomization schedule to allocate women to the prenatal intervention or control group and in a next step to allocate women to a postnatal intervention and

For peer review only - http://bmjopen.bmj.com/site/about/guidelines.xhtml

For peer review only

control group. Per health center (i.e., stratum), women will be individually randomly in permuted blocks of 8 so that, per block, equal numbers are obtained in the prenatal intervention (n=4) and control (n=4) group and equal numbers are also obtained in the postnatal intervention (n=4) and control (n=4) group. The double random sequence will be generated before the start of the study using Stata 15.1 (Statacorp, Texas) by an external research analyst. The allocation group will be coded with two letters (A or B for the prenatal and Y or Z for the postnatal study group) and placed in a sequentially numbered sealed opaque envelopes by project employees, not in direct contact with participants. At study enrollment, the trial midwife will draw the next sealed envelope and allocate the participant to the study group defined by the letter code in the envelope. Blinding of participants and community-based project workers will not be possible since the supplements are identifiable. Field staff responsible for measuring primary and secondary study outcomes are not directly involved in the daily supplementation of the study participants and can therefore be considered to be partially blinded.

For peer review only

Outcomes

Primary outcomes of the RCT

The trial has two primary study outcomes that will be used to assess the impact of the prenatal and the postnatal intervention, respectively:

- Incidence of SGA, defined as birth weight <10th centile of the Intergrowth 21st reference (29);
- Length-for-age z-score (LAZ) calculated using the WHO 2006 growth reference at six months of age (30).

Secondary outcomes of the RCT

A list of the trial's secondary outcomes can be found in Table 2.
Birthweight measurements will be defined using the Intergrowth 21st reference (29) and child anthropometry using the Child Growth Standards developed by the WHO (30).

Table 2 *Secondary outcomes of the RCT on maternal, newborn, and child level*

Maternal outcomes	Newborn	Child
Total and trimester-specific prenatal weight gain and gestational weight change	Birth weight (measured within 72 hours after birth)	Weight-for-Age Z-score at six months of age (WAZ) (and 9 and 12 months on a subsample)

For peer review only - http://bmjopen.bmj.com/site/about/guidelines.xhtml

Probable and possible maternal postnatal depression at 2 and 6 months of child age	Birth length (measured within 72 hours after birth)	Weight-for-Length Z-score at six months of age (WLZ) (and 9 and 12 months on a subsample)
Maternal anemia at the third antenatal consultation	Ponderal or Rohrer's index at birth	Stunting at six months of age
Women's mean and minimum dietary diversity score (measured twice weekly)	Gestational age at delivery	Wasting at six months of age
	Large-for-gestational age	Underweight at six months of age
	Chest circumference (measured within 72 hours after birth)	Duration of exclusive breastfeeding during the first six months of age
	Head circumference (measured within 72 hours after birth)	Incidence of child wasting over first six months of life
	Arm circumference (measured within 72 hours after birth)	Weight gain over first six months of life
	Incidence of preterm birth	Child mortality (between birth and six months of age)
	Fetal loss	Monthly change in LAZ over first six months of life
	Stillbirths	Monthly change in WHZ over first six months of life
		Monthly change in WAZ over first six months of life
		Monthly change in head circumference over first six months of life
		Child morbidity symptoms over first six months of life
		Anemia at six months of age

For peer review only - http://bmjopen.bmj.com/site/about/guidelines.xhtml

		Hemoglobin concentration at six months of age.

Outcomes of the sub-studies

Sub-study 1: Impact of the intervention on neonatal and maternal body composition 2-3 weeks after delivery.

Body composition will be determined in mother-child dyads by deuterium dilution, and analysis of saliva by a Fourier Transform Infrared reader (Agilent FTIR 4500 series). The sub-study will also assess if early-gestation maternal body mass index (defined as body weight in kilograms divided by the square of height in meters) modifies the intervention's effect on neonatal body composition.

Sub-study 2: Impact of the intervention on dietary intake.

A dietary assessment study will be conducted, using 24-hour dietary recalls in a subsample of women. This sub-study will enable us to assess possible substitution of the prenatal diet by the BEP supplement.

Sub-study 3: Impact of the intervention on breastmilk.

Breastmilk samples will be taken in the four study groups to compare the composition and to analyze the interaction between the supplementation periods.

Sample size

With an SGA prevalence of 32% and an anticipated decrease of 7%, a sample of 652 subjects per prenatal arm is required with $\alpha = 0.05$ and $\beta = 0.2$ (31). To accommodate for possible losses, the number of subjects per arm was increased to 888 (total subjects: 1776). Possible losses are based on previous MISAME studies where the prevalence was ~26 %, due to a combination of miscarriage, stillbirths, multifetal pregnancies, out-migrants, maternal deaths and incomplete data (18). For the analysis of an effect of the postnatal intervention on LAZ at six months of age, the minimally detectable effect depends on the presence or absence of an interaction effect between the pre- and postnatal intervention. In the absence of a statistically significant interaction between pre-and postnatal intervention, at sample size of 588 children per postnatal study arm would allow us to detect a

For peer review only - http://bmjopen.bmj.com/site/about/guidelines.xhtml

difference of 0.18 Z-score (SD=1.1), based on a cross-sectional survey conducted in the Gourcy health district in Burkina Faso (32), between study arms with α=0.05 and β=0.20. This implies that if ~1400 singleton live births are available, we allow for a maximum loss to follow-up of 16%. In the presence of a statistically significant interaction between pre-and postnatal intervention, a total sample size of 1176 represents 294 children per factorial combination of the prenatal and postnatal study group (four groups in total). A subgroup size of 294 would allow us to detect a difference in LAZ at six months of age of 0.28 assuming a SD of 1.1, α=0.025 (Bonferroni correction for two primary endpoints analyses) and β=0.20.

Data collection

Anthropometric and clinical procedures

At enrolment, anthropometric measurements from all women will be taken. Gestational age will be determined during an ultrasound consultation by measuring crown-rump length (7-13 weeks), or by calculating the mean of three to four measurements: biparietal diameter, head circumference, abdominal circumference, and femur length (12-26 weeks) (33).

During pregnancy, clinical follow up will consist of antenatal visits following the national guidelines.

At birth, anthropometric measurements of all neonates will be assessed in duplicate within the first 72 hours of life (in practice, the aim is to measure within the 24 hours of life). After birth, mother and child will visit the health care centers monthly for a follow-up on clinical, anthropometric and child morbidity measures (signs including fever, vomiting, diarrhea, cough, difficulty breathing and running nose). A subsample will be visited at home by the project interviewers to collect postnatal data at months 9 and 12.

Hemoglobin concentrations will be measured in women at enrolment and during the 3rd antenatal care visit. This will be conducted at six months of age among children.

Baseline questionnaires

Pre- and postnatal maternal depression will be assessed using the standardized Edinburgh Postnatal Depression Scale (EPDS) questionnaire consisting of 10 questions (34). Study midwives will be trained for this and the questionnaire will be asked at inclusion and at month 2 and 6 after birth. Socio-economic and demographic information from all participants will be collected once included. Trained study interviewers will ask questions on household members' characteristics, household properties, WASH environment, and household food security (35). The women's dietary diversity score will be

For peer review only - http://bmjopen.bmj.com/site/about/guidelines.xhtml

measured in all participating women by the FA during the home visits. This will be enumerated twice a week per participant using the Women's dietary diversity score with 11 food groups (36).

Table 3 shows the overview of the time schedule and measurements of the trial.

Quality of all study data will be insured by a thorough training of all field staff. Procedures to handle data collection tools (questionnaires, anthropometric and clinical measurement material, and laboratory procedures) will be pre-tested in the field during a dry-run of ± three months. Anthropometric measurement standardizations of the field staff will be repeated bimonthly throughout the trial. Anthropometric measurements will be taken in duplicate. Newborns will be measured within 72 hours after birth (preferably within the 24 hours), and all weighing scales and HemoCue 201+ devices will undergo weekly quality control. A WhatsApp group will be set up where problems can be communicated and solved quickly.

All data collection forms of the trial can be found on: *www.misame3.ugent.*

Women will be designated as lost to follow up if they move from the study area or withdraw their participation. Reasons for discontinuation will be recorded.

Women will be enrolled in the study from October 2019 until the total sample size has been reached.

For peer review only

For peer review only - http://bmjopen.bmj.com/site/about/guidelines.xhtml

Table 3 *Participant timeline schedule of enrolment, interventions, assessment and visits.*

	**Enrolment**		**Allocation**	**Post-allocation**											
				**Pregnancy and birth**				**After birth**							
	**Start**	**5-weekly visits**	**ANC 1**	**Household visit**	**Ultra-sound**	**ANC 2, 3 and 4**	**Birth**	**Month 1**	**Month 2**	**Month 3**	**Month 4**	**Month 5**	**Month 6**	**Month 9**	**Month 12**
**Enrolment**															
Census	X														
Pregnancy identification		X													
Pregnancy confirmatory test			X												
Informed consent and allocation to study group			X												
**Study groups**															
Prenatal BEP + IFA			X				X								
Prenatal IFA			X				X								
Postnatal BEP + IFA*							X						X		
Postnatal IFA							X		X						

For peer review only - http://bmjopen.bmj.com/site/about/guidelines.xhtml

**Assessments**															
Mothers															
Baseline questionnaire				X											
Gestational age determination					X										
Skinfold measurements					X										
Weight (kg) and arm circumference (mm) **/***			X			X	X	X	X	X	X	X	X	X	X
Height (cm)			X												
Hemoglobin (g/dL)						X (ANC3)		X							
Women's dietary diversity score (twice weekly)				X											
Maternal depression			X						X				X		
Infants															
Birth weight (kg)							X								
Birth length (cm)							X								
Head circumference (mm)							X	X	X	X	X	X	X		

For peer review only

Chest circumference (mm)							X								
Arm circumference (mm)								X		X			X		X
Morbidity								X	X	X	X	X	X		
Mortality							X	X	X	X	X	X	X		
Weight (kg) and height (cm)								X	X	X	X	X	X	X	X
Breastfeeding practices								X	X	X	X	X	X		
Hemoglobin (g/dL)													X		

* The IFA tablets will be given during the first 6 weeks after birth in the postnatal intervention group, according to the national health protocol.
** Only maternal weight will be taken at birth.
*** In a subsample at month 9 and 12.

For peer review only

Data management and analysis

FAs will use smartphones with computer assisted person interviewing (CAPI) programmed in CSPRO (version 7.3.1) to collect data during household visits. The study data collected by the project medical doctor, project midwives, and interviewers will be done by Survey Solutions data entry software (version 19.12.6) on tablets. This data will be uploaded to a central server on a weekly basis. All questionnaires were programmed and have been tested on the Survey Solutions Designer website and include validation codes to promote the quality of the data entry in the field. Assignments will be sent once a week to the tablets of the field team and preloaded data collected at an earlier contact moment will be used to lower the amount of incorrect data. Paper forms will also be available on the field as a backup.

Further data quality checks will be conducted in Stata 15.1 (Statacorp, Texas). Missing or inconsistent data outliers will be sent back to the field for revision.

Statistical analysis

We refer to the Statistical Analysis Plan (SAP) of the trial; *"Statistical analysis plan: Impact of a prenatal and postnatal balanced energy-protein supplement on birth size and postnatal child growth in Burkina Faso"* published on: *www.misame3.ugent.*

Data monitoring

Data monitoring and auditing

The Data and Safety Monitoring Board (DSMB) is an independent multidisciplinary group whose members are not involved in the trial. The board consists of a Belgian endocrinologist, a Belgian pediatrician, a Burkinabè pediatrician, a Belgian gynecologist, and a Belgian ethicist.

Serious adverse events (SAEs)

FAs will be trained to recognize health issues and will actively refer those participants to see the project midwife in the CSPS in the event they occur. All SAEs will be recorded on a case-by-case basis and verbal autopsies will be conducted for maternal, neonatal, and infant deaths by the field medical doctor.

Patient and public involvement

For peer review only - http://bmjopen.bmj.com/site/about/guidelines.xhtml

For peer review only

MISAME-III has been well accepted by the community, because of the previous positive experiences they had with the MISAME-I and II studies. Through the formative study, women were involved in the choice of BEP supplement. Workshops will be planned at in the end of the study in order to communicate the study results to the community.

Ethics and dissemination

Ethics approval and consent to participate

MISAME-III has been reviewed and approved by the University Hospital of Ghent University (B670201734334) and the Burkinabe ethics (N°2018-22/MS/SG/CM/CEI) committee. Important protocol changes will be noted on *ClinicalTrials.gov*. When eligible women meet the inclusion criteria, project midwives will explain the background and procedure of the complete trial. Written informed consent or assent will be asked from the participating women. In case of illiteracy, a thumb print will be asked and witnessed by the recruiting investigator and one extra witness. Participants will be told that all data collected during the trial is confidential and that they are allowed to withdraw at any time. A copy of the informed consent and assent can be found on: *www.misame3.ugent* and as supplementary file (**Supplementary file 2**).

Confidentiality

A data management plan has been put in place to address concerns regarding the General Data Protection Regulation (GDPR) rules. During the trial, the data files containing personal identifying information will be stored on the Survey Solutions server. Only the principal investigators and the project coordinators will be able to access those files.

Dissemination plan

Upon completion of the trial, all anonymized study data will be available upon request. Final results will be communicated to the participants, the Burkinabè Ministry of health, the field staff, the BMGF, Ghent University researchers and students, AfricSanté, healthcare professionals and other relevant international public institutions. Papers on the study results will be published in peer-reviewed journals and will be available on the project website. All investigators contributing to the realization of the project and publication of results will be included as an author. Other contributors such as the participants, FA and field staff members will be mentioned in the acknowledgments.

Ancillary care

For peer review only - http://bmjopen.bmj.com/site/about/guidelines.xhtml

The MISAME-III project will pay for ancillary care when participants have health issues and in case the costs are not covered by the national health care program. Participants suffering harm due to their trial participation will be covered.

Discussion

In this paper the protocol of an individually randomized 4-arm efficacy trial in rural Burkina Faso has been described in which pregnant and lactating women in the intervention group will receive a BEP supplement together with IFA tablets. The control group will only receive the standard IFA treatment.

The key features of the present trial are first, the inclusion of a formative study for a better understanding of which type of supplement is preferred, what taste is most acceptable, and which factors affect adherence in the study population. Second, the supplementation will not only be given during pregnancy but also during the first six months after birth. This will give us the opportunity to assess the specific value of postnatal supplementation on several outcomes. Third, the observed daily intake of intervention and control supplements is a key feature to ensure compliance and to avoid sharing of the supplements with other household members. Fourth, MISAME-III has the advantage of being the third trial of its kind in the study area. This presents an opportunity to anticipate the issues that arose in previous trials. For instance, women in specific villages tended to leave their homes for a longer period to go work on the fields outside the village. This posed problems in the continuation of the supplementation in the past and will be taken into consideration during MISAME-III. Fourth, four sub-studies are nested in the main trial that will provide insight into the mechanism by which prenatal BEP supplementation affects birth and infant outcomes. And last, similar studies are being conducted in other countries, allowing for comparison between results from different contexts.

The MISAME III study will provide evidence on the impact of BEP supplements on birth and infant size using a rigorous study design. The study results will further strengthen and refine WHO's recommendation on the use of context-specific BEP supplementation during pregnancy and lactation.

Abbreviations

AE; Adverse Events, ANC; Antenatal Care, BEP; Balanced Energy-Protein Supplement, BMGF; Bill and Melinda Gates Foundation, BMI; Body Mass Index, CSPro; Census and Survey Processing System, CSPS; Centre de Santé et de Promotion Social, DHS; Demographic and Health Survey, DSMB; Data and Safety Monitoring Board, EP(D)S; Edinburgh Postnatal (Postpartum) Depression Scale, FA; Femme Accompagnante, GDPR; General Data Protection Regulation, HDI; Human Development Index, IFA;

For peer review only - http://bmjopen.bmj.com/site/about/guidelines.xhtml

Iron/Folic Acid, IUGR; Intra Uterine Growth Restriction, LAZ; Length-for-Age Z-score, LBW; Low Birth Weight, LMIC; Low and Middle Income Countries, LMP; Last Menstrual Period, LQAS; Lot Quality Assessment Sampling, MISAME; MIcronutriments pour la SAnté de la Mère et de l'Enfant, MMS; Multiple-Micronutrient Supplements, RCT; Randomized Controlled Trial, SAE; Serious Adverse Events, SGA; Small-for-Gestational Age, UNICEF; United Nations International Children's Emergency Fund, UNIMMAP; UNICEF/WHO/UNU International Multiple Micronutrient, WASH; water, sanitation and hygiene, WAZ; Weight-for-Age Z-score, WHO; World Health Organization, WHZ; Weight-for-Height Z-score, WLZ; Weight-for-Length Z-score.

Acknowledgements

The MISAME Study Group thanks the pregnant women and their families for the time spent in this study. We would like to acknowledge the staff of AFRICSanté; including Henri Somé for his support on the CAPI software, the field medical Dr. Anderson Compaoré, the data collectors (midwives, interviewers and femmes accompagnantes), Dr. Alain Hein for the standardization exercises and Dr. Hermann Lanou for his support during the training. We would like to thank Dr. Sheila Isanaka for her collaboration during the formative research. Our private sector partner Nutriset (France) is acknowledged for donating the BEP supplements.

For peer review only

Competing interests statement

KV: none to declare
BdK: none to declare
LCT: none to declare
NDC: none to declare
MO: none to declare
TDC: none to declare
GHC: none to declare
RG: none to declare
CL: none to declare
LH: none to declare
PK: none to declare

Contributor Statement

KV wrote the manuscript;
PK, LH, CL, NDC, LCT, KV, BdK, TDC, GHC designed the study and the protocol;
PK, LH, LCT, KV, BdK designed the study material tools;
LCT, LH, KV, BdK, MO, RG trained the field data collectors;
TDC, GHC critically reviewed and revised the manuscript;
All authors contributed substantially to the manuscript and approved the final version.

Correspondence

Katrien.Vanslambrouck@UGent.be
katvsb@hotmail.com

Funding Information

This work was funded by the Bill and Melinda Gates Foundation. Grant number OPP1175213.

The trial is registered on Clinical Trials.gov (identifier: NCT03533712) prior to recruitment. Enrolment started in October 2019.

Statement
Nutriset will not be involved in the design, the implementation, the analysis and the write-up of the results.
The BMGF will not be involved in the design, the implementation, the analysis and the write-up of the results.

REFERENCES

1. Lee AC, Katz J, Blencowe H, Cousens S, Kozuki N, Vogel JP, et al. National and regional estimates of term and preterm babies born small for gestational age in 138 low-income and middle-income countries in 2010. The Lancet Global Health. 2013;1(1):e26-e36.
2. Katz J, Lee AC, Kozuki N, Lawn JE, Cousens S, Blencowe H, et al. Mortality risk in preterm and small-for-gestational-age infants in low-income and middle-income countries: a pooled country analysis. The Lancet. 2013;382(9890):417-25.
3. van Rossum CTM, Fransen HP, Verkaik-Kloosterman J, Buurma-Rethans EJM, Ocké MC. Dutch National Food Consumption Survey 2007–2010: Diet of children and adults aged 7 to 69 years2011.
4. Lee AC, Kozuki N, Cousens S, Stevens GA, Blencowe H, Silveira MF, et al. Estimates of burden and consequences of infants born small for gestational age in low and middle income countries with INTERGROWTH-21st standard: analysis of CHERG datasets. bmj. 2017;358.
5. Stevens B, Buettner P, Watt K, Clough A, Brimblecombe J, Judd J. The effect of balanced protein energy supplementation in undernourished pregnant women and child physical growth in low-and middle-income countries: a systematic review and meta-analysis. Maternal & child nutrition. 2015;11(4):415-32.
6. Organization WH. WHO recommendations on antenatal care for a positive pregnancy experience: World Health Organization; 2016.
7. Krishna U, Bhalerao S. Placental insufficiency and fetal growth restriction. The Journal of Obstetrics and Gynecology of India. 2011;61(5):505-11.
8. Mridula D, Mishra C, Chakravorty A. Dietary intake of expectant mother. Indian J Nutr Diet. 2003;40(1):24-30.
9. Tang AM, Chung M, Dong K, Terrin N, Edmonds A, Assefa N, et al. Determining a global mid-upper arm circumference cutoff to assess malnutrition in pregnant women. Food and Nutrition Technical Assistance. 2016.
10. Branca F, Grummer-Strawn L, Borghi E, Blössner Md, Onis Md. Extension of the WHO maternal, infant and young child nutrition targets to 2030. SCN News. 2015(41):55-8.
11. Keats EC, Haider BA, Tam E, Bhutta ZA. Multiple-micronutrient supplementation for women during pregnancy. Cochrane Database of Systematic Reviews. 2019(3).
12. Hambidge KM, Westcott JE, Garcés A, Figueroa L, Goudar SS, Dhaded SM, et al. A multicountry randomized controlled trial of comprehensive maternal nutrition supplementation initiated before conception: the Women First trial. The American journal of clinical nutrition. 2019;109(2):457-69.
13. Ota E, Hori H, Mori R, Tobe-Gai R, Farrar D. Antenatal dietary education and supplementation to increase energy and protein intake. Cochrane Database of Systematic Reviews. 2015(6).
14. Imdad A, Bhutta ZA. Maternal nutrition and birth outcomes: effect of balanced protein-energy supplementation. Paediatr Perinat Epidemiol. 2012;26 Suppl 1:178-90.
15. Kramer MS, Kakuma R. Energy and protein intake in pregnancy. Cochrane Database of systematic reviews. 2003(4).

For peer review only - http://bmjopen.bmj.com/site/about/guidelines.xhtml

16. Bill and Melinda Gates Foundation (BMGF). Framework and Specifications for the Nutritional Composition of a Food Supplement for Pregnant and Lactating Women (PLW) in Undernourished and Low-Income Settings. Expert consultation Seatle, US; 2016 2016.
17. Roberfroid D, Huybregts L, Lanou H, Henry M-C, Meda N, Menten J, et al. Effects of maternal multiple micronutrient supplementation on fetal growth: a double-blind randomized controlled trial in rural Burkina Faso–. The American journal of clinical nutrition. 2008;88(5):1330-40.
18. Huybregts L, Roberfroid D, Lanou H, Menten J, Meda N, Van Camp J, et al. Prenatal food supplementation fortified with multiple micronutrients increases birth length: a randomized controlled trial in rural Burkina Faso. Am J Clin Nutr. 2009;90(6):1593-600.
19. United Nations Development Programme. Human Development Reports [Internet] 2019. Available from: http://hdr.undp.org/en/countries/profiles/BFA.
20. Unicef. The state of the world's children: children with disabilities: UN; 2013.
21. INSD MD, Macro I. Enquête démographique et de santé et à indicateurs multiples (EDSBF-MICS IV), Rapport préliminaire, Burkina Faso, 2010. Ouagadougou: INSD. 2011;40.
22. Institut National de la Statistique et de la Démographie (INSD) et ICF International. Enquête Démographique et de Santé et à Indicateurs Multiples du Burkina Faso 2010. Calverton, Maryland, USA : INSD et ICF International. 2012.
23. MOH. National Iodine Status and Anemia Survey, Burkina Faso. 2014.
24. MOH. MdlS. Enquête Nutritionnelle Nationale 2013. 2013.
25. Savy M, Martin-Prével Y, Sawadogo P, Kameli Y, Delpeuch F. Use of variety/diversity scores for diet quality measurement: relation with nutritional status of women in a rural area in Burkina Faso. European journal of clinical nutrition. 2005;59(5):703.
26. Huybregts LF, Roberfroid DA, Kolsteren PW, Van Camp JH. Dietary behaviour, food and nutrient intake of pregnant women in a rural community in Burkina Faso. Maternal & child nutrition. 2009;5(3):211-22.
27. Jones L, de Kok B, Moore K, de Pee S, Bedford J, Vanslambrouck K, et al. Acceptability of 12 fortified balanced energy protein supplements-Insights from Burkina Faso. Maternal & Child Nutrition. 2020:e13067.
28. de Kok BM, K., Jones, L., Vanslambrouck, K., Toe, L.C., Ouedraogo, M., Ganaba, R., de Pee, S., Bedford, J., Lachat, C., Kolsteren, P., Isanaka, S. . Acceptability and at-home consumption of two fortified balanced energy-protein supplements among pregnant women in rural Burkina Faso: a mixed methods study. Maternal & Child Nutrition, manuscript in production 2020.
29. Villar J, Altman D, Purwar M, Noble J, Knight H, Ruyan P, et al. The objectives, design and implementation of the INTERGROWTH-21st Project. BJOG: An International Journal of Obstetrics & Gynaecology. 2013;120:9-26.
30. de Onis M, Branca F. Childhood stunting: a global perspective. Matern Child Nutr. 2016;12 Suppl 1:12-26.
31. Huybregts LF, Roberfroid DA, Kolsteren PW, Van Camp JH. Dietary behaviour, food and nutrient intake of pregnant women in a rural community in Burkina Faso. Matern Child Nutr. 2009;5(3):211-22.
32. Becquey E, Huybregts L, Zongrone A, Le Port A, Leroy JL, Rawat R, et al. Impact on child acute malnutrition of integrating a preventive nutrition package into facility-based screening for acute malnutrition during well-baby consultation: A cluster-randomized controlled trial in Burkina Faso. PLoS medicine. 2019;16(8).
33. Salomon L, Alfirevic Z, Da Silva Costa F, Deter R, Figueras F, Ghi T, et al. ISUOG Practice Guidelines: ultrasound assessment of fetal biometry and growth. Ultrasound in Obstetrics & Gynecology. 2019;53(6):715-23.
34. Cox JL, Holden JM, Sagovsky R. Detection of postnatal depression: development of the 10-item Edinburgh Postnatal Depression Scale. The British journal of psychiatry. 1987;150(6):782-6.
35. Femi A. Perception of performance appraisal and workers' performance in Wema Bank Headquarters, Lagos. Global Journal of Arts, Humanities and Social Sciences. 2013;1(4):89-101.

For peer review only - http://bmjopen.bmj.com/site/about/guidelines.xhtml

36. Martin-Prével Y, Allemand P, Wiesmann D, Arimond M, Ballard T, Deitchler M, et al. Moving forward on choosing a standard operational indicator of women's dietary diversity. FAO; 2015.

Figure legend

Figure 1 : Study design of the RCT

For peer review only

Figure 1 *Study design of the RCT*

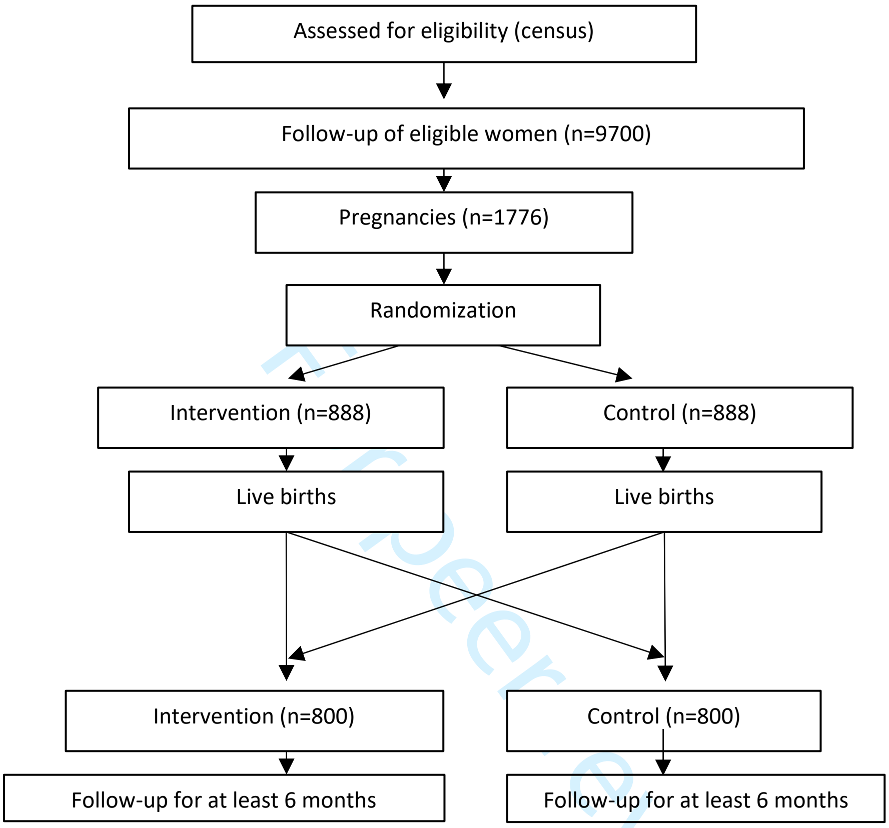

For peer review only

Supplementary file : SPIRIT Checklist 2013.

Reporting checklist for protocol of a clinical trial.

Based on the SPIRIT guidelines.

Instructions to authors

Complete this checklist by entering the page numbers from your manuscript where readers will find each of the items listed below.

Your article may not currently address all the items on the checklist. Please modify your text to include the missing information. If you are certain that an item does not apply, please write "n/a" and provide a short explanation.

Upload your completed checklist as an extra file when you submit to a journal.

In your methods section, say that you used the SPIRITreporting guidelines, and cite them as:

Chan A-W, Tetzlaff JM, Altman DG, Laupacis A, Gøtzsche PC, Krleža-Jerić K, Hróbjartsson A, Mann H, Dickersin K, Berlin J, Doré C, Parulekar W, Summerskill W, Groves T, Schulz K, Sox H, Rockhold FW, Rennie D, Moher D. SPIRIT 2013 Statement: Defining standard protocol items for clinical trials. Ann Intern Med. 2013;158(3):200-207

		Reporting Item	Page Number
**Administrative information**			
Title	#1	Descriptive title identifying the study design, population, interventions, and, if applicable, trial acronym	1
Trial registration	#2a	Trial identifier and registry name. If not yet registered, name of intended registry	2
Trial registration: data set	#2b	All items from the World Health Organization Trial Registration Data Set	n/a
Protocol version	#3	Date and version identifier	1
Funding	#4	Sources and types of financial, material, and other support	20
Roles and responsibilities: contributorship	#5a	Names, affiliations, and roles of protocol contributors	1 / 20
Roles and responsibilities: sponsor contact information	#5b	Name and contact information for the trial sponsor	20

For peer review only

Roles and responsibilities: sponsor and funder	#5c	Role of study sponsor and funders, if any, in study design; collection, management, analysis, and interpretation of data; writing of the report; and the decision to submit the report for publication, including whether they will have ultimate authority over any of these activities	20
Roles and responsibilities: committees	#5d	Composition, roles, and responsibilities of the coordinating centre, steering committee, endpoint adjudication committee, data management team, and other individuals or groups overseeing the trial, if applicable (see Item 21a for data monitoring committee)	17
**Introduction**			
Background and rationale	#6a	Description of research question and justification for undertaking the trial, including summary of relevant studies (published and unpublished) examining benefits and harms for each intervention	4
Background and rationale: choice of comparators	#6b	Explanation for choice of comparators	4
Objectives	#7	Specific objectives or hypotheses	4
Trial design	#8	Description of trial design including type of trial (eg, parallel group, crossover, factorial, single group), allocation ratio, and framework (eg, superiority, equivalence, non-inferiority, exploratory)	5
**Methods: Participants, interventions, and outcomes**			
Study setting	#9	Description of study settings (eg, community clinic, academic hospital) and list of countries where data will be collected. Reference to where list of study sites can be obtained	6
Eligibility criteria	#10	Inclusion and exclusion criteria for participants. If applicable, eligibility criteria for study centres and individuals who will perform the interventions (eg, surgeons, psychotherapists)	6
Interventions: description	#11a	Interventions for each group with sufficient detail to allow replication, including how and when they will be administered	8

For peer review only

Interventions: modifications	#11b	Criteria for discontinuing or modifying allocated interventions for a given trial participant (eg, drug dose change in response to harms, participant request, or improving / worsening disease)	n/a
Interventions: adherance	#11c	Strategies to improve adherence to intervention protocols, and any procedures for monitoring adherence (eg, drug tablet return; laboratory tests)	7 /
Interventions: concomitant care	#11d	Relevant concomitant care and interventions that are permitted or prohibited during the trial	n/a
Outcomes	#12	Primary, secondary, and other outcomes, including the specific measurement variable (eg, systolic blood pressure), analysis metric (eg, change from baseline, final value, time to event), method of aggregation (eg, median, proportion), and time point for each outcome. Explanation of the clinical relevance of chosen efficacy and harm outcomes is strongly recommended	9
Participant timeline	#13	Time schedule of enrolment, interventions (including any run-ins and washouts), assessments, and visits for participants. A schematic diagram is highly recommended (see Figure)	14
Sample size	#14	Estimated number of participants needed to achieve study objectives and how it was determined, including clinical and statistical assumptions supporting any sample size calculations	11
Recruitment	#15	Strategies for achieving adequate participant enrolment to reach target sample size	6
**Methods: Assignment of interventions (for controlled trials)**			
Allocation: sequence generation	#16a	Method of generating the allocation sequence (eg, computer-generated random numbers), and list of any factors for stratification. To reduce predictability of a random sequence, details of any planned restriction (eg, blocking) should be provided in a separate document that is unavailable to those who enrol participants or assign interventions	8
Allocation concealment mechanism	#16b	Mechanism of implementing the allocation sequence (eg, central telephone; sequentially numbered, opaque, sealed envelopes), describing any steps to conceal the sequence until interventions are assigned	8

For peer review only

Allocation: implementation	#16c	Who will generate the allocation sequence, who will enrol participants, and who will assign participants to interventions	8
Blinding (masking)	#17a	Who will be blinded after assignment to interventions (eg, trial participants, care providers, outcome assessors, data analysts), and how	9
Blinding (masking): emergency unblinding	#17b	If blinded, circumstances under which unblinding is permissible, and procedure for revealing a participant's allocated intervention during the trial	9
**Methods: Data collection, management, and analysis**			
Data collection plan	#18a	Plans for assessment and collection of outcome, baseline, and other trial data, including any related processes to promote data quality (eg, duplicate measurements, training of assessors) and a description of study instruments (eg, questionnaires, laboratory tests) along with their reliability and validity, if known. Reference to where data collection forms can be found, if not in the protocol	12
Data collection plan: retention	#18b	Plans to promote participant retention and complete follow-up, including list of any outcome data to be collected for participants who discontinue or deviate from intervention protocols	13
Data management	#19	Plans for data entry, coding, security, and storage, including any related processes to promote data quality (eg, double data entry; range checks for data values). Reference to where details of data management procedures can be found, if not in the protocol	17
Statistics: outcomes	#20a	Statistical methods for analysing primary and secondary outcomes. Reference to where other details of the statistical analysis plan can be found, if not in the protocol	17
Statistics: additional analyses	#20b	Methods for any additional analyses (eg, subgroup and adjusted analyses)	n/a
Statistics: analysis population and missing data	#20c	Definition of analysis population relating to protocol non-adherence (eg, as randomised analysis), and any statistical methods to handle missing data (eg, multiple imputation)	17
**Methods: Monitoring**			

For peer review only

Data monitoring: formal committee	#21a	Composition of data monitoring committee (DMC); summary of its role and reporting structure; statement of whether it is independent from the sponsor and competing interests; and reference to where further details about its charter can be found, if not in the protocol. Alternatively, an explanation of why a DMC is not needed	17
Data monitoring: interim analysis	#21b	Description of any interim analyses and stopping guidelines, including who will have access to these interim results and make the final decision to terminate the trial	n/a
Harms	#22	Plans for collecting, assessing, reporting, and managing solicited and spontaneously reported adverse events and other unintended effects of trial interventions or trial conduct	17
Auditing	#23	Frequency and procedures for auditing trial conduct, if any, and whether the process will be independent from investigators and the sponsor	n/a
**Ethics and dissemination**			
Research ethics approval	#24	Plans for seeking research ethics committee / institutional review board (REC / IRB) approval	18
Protocol amendments	#25	Plans for communicating important protocol modifications (eg, changes to eligibility criteria, outcomes, analyses) to relevant parties (eg, investigators, REC / IRBs, trial participants, trial registries, journals, regulators)	18
Consent or assent	#26a	Who will obtain informed consent or assent from potential trial participants or authorised surrogates, and how (see Item 32)	18
Consent or assent: ancillary studies	#26b	Additional consent provisions for collection and use of participant data and biological specimens in ancillary studies, if applicable	n/a
Confidentiality	#27	How personal information about potential and enrolled participants will be collected, shared, and maintained in order to protect confidentiality before, during, and after the trial	18
Declaration of interests	#28	Financial and other competing interests for principal investigators for the overall trial and each study site	20

For peer review only

Data access	#29	Statement of who will have access to the final trial dataset, and disclosure of contractual agreements that limit such access for investigators	18
Ancillary and post trial care	#30	Provisions, if any, for ancillary and post-trial care, and for compensation to those who suffer harm from trial participation	18
Dissemination policy: trial results	#31a	Plans for investigators and sponsor to communicate trial results to participants, healthcare professionals, the public, and other relevant groups (eg, via publication, reporting in results databases, or other data sharing arrangements), including any publication restrictions	18
Dissemination policy: authorship	#31b	Authorship eligibility guidelines and any intended use of professional writers	18
Dissemination policy: reproducible research	#31c	Plans, if any, for granting public access to the full protocol, participant-level dataset, and statistical code	n/a
**Appendices**			
Informed consent materials	#32	Model consent form and other related documentation given to participants and authorised surrogates	18
Biological specimens	#33	Plans for collection, laboratory evaluation, and storage of biological specimens for genetic or molecular analysis in the current trial and for future use in ancillary studies, if applicable	n/a

The SPIRIT checklist is distributed under the terms of the Creative Commons Attribution License CC-BY-ND 3.0. This checklist was completed on 06. March 2020 using https://www.goodreports.org/, a tool made by the EQUATOR Network in collaboration with Penelope.ai

For peer review only

1. PARTICIPANT INFORMATION SHEET

THE EFFECT OF BALANCED ENERGY-PROTEIN SUPPLEMENTATION DURING PREGNANCY AND LACTATION ON BIRTH OUTCOMES AND INFANT GROWTH IN RURAL BURKINA FASO

Coordinating Investigator : Prof. Dr. Patrick Kolsteren
Principal Investigator: Dr. Laeticia Celine TOE
Sponsor of the study: Ghent University
Participant Number: |__||__||__||__||__| _ |__||__|

For peer review only

Dear madam,

You are invited to participate in a study that wants to study the effect of providing a food supplement during pregnancy and lactation on the growth of your baby. The study is a collaboration between the Institut de Recherche en Sciences de la Santé (IRSS) and AfricSanté in Burkina Faso, Ghent University of Belgium and the International Food Policy Research Institute (IFPRI) in the USA. Before you decide to participate in this study, it is important that you read/ are read and explained to this form, because it explains your rights and our responsibilities to you. In this information and consent form, the purpose, examinations, advantages, risks and inconveniences related to this study are explained. The available alternatives and the right to withdraw your consent to participate at any time are also described below. No promises or guarantees can be made about the results of the study. You have the right to ask questions at any time, for example about the possible and/or known risks related to this study.

PURPOSE AND DESCRIPTION OF THE STUDY

This research study will provide more evidence and insight into how we can improve pregnancy outcomes , birthweight of babies and the growth of newborns. With this study, we aim to assess whether the provision of the food supplement under investigation can improve the growth of fetuses during pregnancy and that of infants during their first 6 months of life. In addition, we will evaluate how the food supplements that are offered to you improve the nutritional quality of the human milk produced for your child, as well as your and your child's body composition.

We will thus compare the effect of 2 different approaches. One is to give you a tablet during your pregnancy and with the other approach participants will also receive a food supplement next to the tablet. The tablet is what you normally received for any pregnancy according to the guidelines of the ministry of health of Burkina Faso. We will provide the supplement for the entire duration of your pregnancy. After birth some mothers will continue to receive a food supplement for six months. Who receives what is entirely determined by chance. In total, 1776 pregnant women living in the catchment area of the health centers of de Boni, Dohoun, Dougmato II, Karaba, Kari and Koumbia will be recruited to participate in this study.

For peer review only - http://bmjopen.bmj.com/site/about/guidelines.xhtml

How the study is done

If you are confirmed pregnant and accept to participate in this study, the midwife will do a full medical check-up of you and your baby according to medical standards. She will also inform you about a good dietary practices for you during your pregnancy.
After the check-up we will provide you either with the tablets, or the food supplements.

Thereafter, you will receive a daily visit from the community health worker who will ask you to take the tablet or eat the food supplement. A ultrasound examination will be planned for you within 2 weeks after your first consultation and will be performed by the project doctor. We will also arrange for you regular check-ups of your pregnancy by the midwife or the medical doctor.

Examinations in the context of the study

If you accept to participate in the study and if you and your child meet all the conditions for participating in the study, the following tests and examinations will be performed:

- We will take your weight t and height and ask you your age at inclusion.
- The midwife will invite you for antenatal check-ups according to the guidelines of the ministry of health and the medical doctor will perform an echography of your baby. This is not invasive and has no known risks. It is like taking a picture of your baby. As part of the routine check-up we will take fingerpick blood to test whether you are anemic and give you treatment when necessary.
- We will also ask you questions about your mental wellbeing, e.g. how you are living your pregnancy and if something bothers you in your everyday life.
- After delivery we will again take fingerpick blood to test whether you are anemic and give you treatment when necessary.
- When you deliver we will weigh your baby and measure his/her length and head and chest circumferences.
- After birth we will visit you or ask you to come to the health center every month to have the weight and the length of you baby taken. At that time we will also ask you question on illnesses your child might have had in the previous week and on what he/she has been eating.
- We will also perform a finger/heel prick on your baby to see if he/she is anemic and a treatment will be provided if necessary
- Halfway through pregnancy in some of the participating women, we will ask you questions about what and how much they have eaten the day before the interview. This should take about half an hour.

When the doctor identifies a medical problem he will see to it that you receive the appropriate information and necessary treatment, and will refer you when necessary.

If your child suffers from a disease or undernutrition, he/she will be treated in the best way possible. In such case, your child will be referred to the local health center or the district hospital, for further physical examination by a medical doctor.

Voluntary participation

You participate entirely voluntarily in this study. You have the right to refuse to participate in the study. Your decision to participate or not in this study, or to stop your participation in this study will have no influence whatsoever on present and future medical consultations and possible treatments. You also have the right yourself to stop your participation in the study at any time, even after you have signed

For peer review only - http://bmjopen.bmj.com/site/about/guidelines.xhtml

this informed consent form. The withdrawal of your consent will not cause any disadvantage or loss of advantages / privileges.

RISKS AND INCONVENIENCES

Finger-prick blood will be taken from you at the first ante-natal consultation. You will experience a prick in your finger, however the prick is not invasive. Antiseptic measures will be taken to prevent any inflammation/ infection of your finger.

The food supplement used in this study is safe for you and your baby. The food supplement contains milk, sugar, oil, peanut butter and a mix of vitamins. Previous studies in Houndé that provided similar food supplements during pregnancy have not documented any complications during pregnancy or delivery. However, the supplement contains milk, which can cause bloating, flatulence and other digestive discomfort in some people. We encourage you to notify us, should you experience these effects, so we can take measures to insure you the best comfort possible.

All other investigation are routinely done as part of the follow-up of pregnancy.

Some of you will be asked to donate a small amount of breast milk at two time points during the study (between 1-2 months and 3-4 months age of your child). This will not diminish the amount of milk for your child or influence lactating performance. We will ask your permission for this donation again when the time comes.

In a random sample of all participants, we will assess body composition after delivery in the mother and the infant, using a special water that has been proven safe for such use. If you are chosen for that examination, we will provide detailed explanations of the procedure to you and a specific consent will be ask before we perform the assessment. Your privacy will be respected at all times

BENEFITS

We expect to show that taking food supplements during pregnancy and lactation will help children grow better and improve the quality of breastmilk. If this would be the case we will have the possibility to change policy to provide supplements to pregnant and lactating women.

PROTECTION OF YOUR PRIVATE LIFE

Your identity and your participation in this study will be treated strictly confidential. The specific information we obtain from you will not be shared with anybody, except the study investigators and partner institutes. Your identity remains secret since your personal information will only be designated by a unique participant number. Your name will not appear in any reports or publication resulting from this study. After the study is completed, you may request information about the study results. As soon as possible (maximum 5 years) after the study is completed, all personal information from participant shall be deleted from all databases to ensure complete anonymity. Those anonymized databases shall be shared with other researchers to advance research on mother and child health. This shall be done in strict accordance with international laws and regulations about privacy.

ETHICS COMMITTEE

Before starting, this study has been reviewed and approved by an independent Ethics Committee in Belgium, namely the Ethics Committee of the University Hospital in Ghent and it has been reviewed and approved by the Ethics Committee Centre Muraz in Burkina Faso. These committees also perform

For peer review only - http://bmjopen.bmj.com/site/about/guidelines.xhtml

continuous reviews of the study during its progression to make sure the study is carried out in the safest possible way.

COMPENSATIONS AND INCIDENTS THAT MAY OCCUR IN THIS STUDY

All costs related to sickness occurring during the study will be reimbursed to you. Investigators shall seek to provide a compensation and/or the best possible treatment in the event of damages/ injuries that may occur as a result of your participation in this study. For any other damages thought to be related to your participation in this study, and occuring after the study has been completed, participant may file a complaint to the relevant jurisdiction, which will treated in accordance with applicable laws in Burkina Faso.

CONTACT PERSONS IN CASE YOU HAVE QUESTIONS ABOUT THIS STUDY

If you have any questions concerning your participation in this study, or think you have been injured as a result of the study or have a reaction to the study food, inform the village health worker who visits you. S/he will bring you immediately in contact with the project coordinator or the project doctor. All village health workers have cell phones to contact these persons directly. You may also contact, now, during or after the study:

The principle investigator in Burkina Faso: Dr. Laeticia Celine Toe, Tel : +226 71 00 72 72
The president of the ethic committee: Dr. Adama Dembélé, Tel: +226 20 97 01 02
Or The local project Coordinator : Mr. Moctar Ouédraogo, Tel: +226 70 23 81 98
Or project medical doctor: Compaoré S. Anderson Casimir Tel : +226 70 57 51 02

For peer review only

Informed consent

Before you agree to participate in the informal consensus activity, you need to be aware that:

- This study was presented and cleared by the Ethical Review Board of the Ghent University and the ethics committee of Centre Muraz, Burkina Faso.
- This clearance is not to be taken as an obligation to take part in this study.
- Your participation is only voluntary and will be confirmed by signing this form. If you wish, you can withdraw from this study at any point, even after signing this form; you can withdraw by contacting the researcher (below) through email or telephone. You do not have to motivate or explain the decision of withdrawal. In case of withdrawal, your data already collected will be used to assess study outcomes.
- You can revise your answers to the questions if you wish so.
- Your input will be stored anonymously; researchers not involved in the data collection will not have access to your personal data and name. Anonymized databases shall be shared with other researchers for to advance research on maternal and child health.
- You can contact the researcher or the coordinator of the project at any time if you wish to obtain more information regarding this study
- There are very limited risks related to your participation in this study. Those are mentioned under the section " risks and inconveniences" of the information sheet. However, in accordance with the Directive concerning experiments on humans (07/05/2004), a civil liability insurance has been foreseen in the event of any injury or damage occurring and deemed due to your participation in this study.

For peer review only

Part intended only for the participant

I, the undersigned, ____
(name and surname) confirm that I have been informed about the MISAME-III clinical trial and that I have received a copy of the Participant Information Sheet and a copy of the consent form. I confirm that I have read and / or understood the four pages of the information sheet for participants. The study responsible gave me enough information about the conditions and duration of the study, and the possible risks and disadvantages. In addition, I had enough time to review the information and to ask questions, and I received satisfactory answers.

- I understand that I can terminate my participation in this study at any time after notifying the study responsible and this decision will not cause any inconvenience to me or my child.
- I am aware of the purposes for which the data is collected, processed and used in this study.
- I am ready to give information about my medical history and that of my child, or about any medication taken or participation in another study.
- I voluntarily consent to participate in this study and to cooperate with the required examinations and tests.
- I consent to my data being shared anonymously for the benefit of research and maternal and child health.
- I understand that auditors, employee representatives, the Commission for Medical Ethics or competent authorities may inspect my data in order to verify the information collected. By signing this document, I give permission for this control. At all times my privacy will be respected.
- I understand that the biological samples collected from me and / or my child will be sent to Europe or the USA for analysis, and this in strict respect of my confidentiality.
- I am aware that this study has been approved by an independent commission for medical ethics linked to the University Hospital of Ghent, examined by the Ethics Committee of the Center Muraz in Burkina Faso and that this study will be carried out in accordance with the guidelines Good Clinical Practice Guidelines and the Declaration of Helsinki, established for the protection of people participating in clinical trials.

Signature (or fingerprint) of the participant

____

For peer review only - http://bmjopen.bmj.com/site/about/guidelines.xhtml

In case of minority

Informed consent of the legal representative

I, the undersigned, ____ (name and surname), legal representative of ____ (name and surname) confirm that I have been informed about the MISAME-III clinical trial and that I have received a copy information sheet for participants and a copy of the consent form. I confirm that I have read and / or understood the 6 pages of the information sheet for participants. The study responsible gave me enough information about the conditions and duration of the study, the procedures, the advantages and possible disadvantages. In addition, I had enough time to review the information and to ask questions, and received satisfactory answers.

- I understand that I can withdraw my consent at any time after having informed the study managers and this decision will not cause any inconvenience to my child / wife / ward or to myself.
- I am aware of the purposes for which the data is collected, processed and used in this study.
- I voluntarily consent to my child / wife / ward participating in this study.
- I understand that auditors, employee representatives, the Commission for Medical Ethics or competent authorities may inspect their data in order to verify the information collected. By signing this document, I give permission for this control. At all times his privacy will be respected.

Date: | ___ | ___ | / | ___ | ___ | / | ___ | ___ | ___ | ___ | (dd / mm / yyyy)

Signature (or fingerprint) of the participant's legal representative

____

For peer review only - http://bmjopen.bmj.com/site/about/guidelines.xhtml

Part intended for the investigator

I, the undersigned, ____ confirm that I have informed, ____

(full name of the participant) and that she has:

Consent to participate in the study ☐

Refused to participate in the study ☐

Reason for refusal (mark not filled in if no reason provided)

____

____

Date | ___ | ___ | / | ___ | ___ | / | ___ | ___ | ___ | ___ | (dd / mm / yyyy)

Signature: ____

If the participant is unable to read and / or write, an impartial witness should be present during the consent discussion. After having read and explained the information sheet and the informed consent form to the participant. After she has verbally consented to her participation in the study, and has affixed her fingerprint, the witness should complete the name of the participant, add the date, and personally sign and date the consent form. By signing the consent form, the witness certifies that the information in the information sheet and the consent form have been precisely explained and understood by the participant and that the participant has freely given her consent.

Name and first name (s) of witness:

____

Signature of witness :

____

Date: | ___ | ___ | / | ___ | ___ | / | ___ | ___ | ___ | ___ | (dd / mm / yyyy)

For peer review only

Contact Principal investigator

TOE Laeticia Celine
IRSS/DRO site Ecole Jamot
01 BP 545 Bobo-Dioulasso
Tél: +226 71 00 72 72
Email: cellaety@yahoo.fr

Contact of project medical doctor
Compaoré S. Anderson Casimir
Tel : +226 70 57 51 02
Email : discomp4523@gmail.com

For peer review only
